# Evidence for a Transketolase-Mediated Metabolic Checkpoint Governing Biotrophic Growth in Rice Cells by the Blast Fungus *Magnaporthe oryzae*

**DOI:** 10.1371/journal.ppat.1004354

**Published:** 2014-09-04

**Authors:** Jessie Fernandez, Margarita Marroquin-Guzman, Richard A. Wilson

**Affiliations:** Department of Plant Pathology, University of Nebraska-Lincoln, Lincoln, Nebraska; Oregon State University, United States of America

## Abstract

The blast fungus *Magnaporthe oryzae* threatens global food security through the widespread destruction of cultivated rice. Foliar infection requires a specialized cell called an appressorium that generates turgor to force a thin penetration hypha through the rice cuticle and into the underlying epidermal cells, where the fungus grows for the first days of infection as a symptomless biotroph. Understanding what controls biotrophic growth could open new avenues for developing sustainable blast intervention programs. Here, using molecular genetics and live-cell imaging, we dismantled *M. oryzae* glucose-metabolizing pathways to reveal that the transketolase enzyme, encoded by *TKL1*, plays an essential role in facilitating host colonization during rice blast disease. In the absence of transketolase, Δ*tkl1* mutant strains formed functional appressoria that penetrated rice cuticles successfully and developed invasive hyphae (IH) in rice cells from primary hyphae. However, Δ*tkl1* could not undertake sustained biotrophic growth or cell-to-cell movement. Transcript data and observations using fluorescently labeled histone H1:RFP fusion proteins indicated Δ*tkl1* mutant strains were alive in host cells but were delayed in mitosis. Mitotic delay could be reversed and IH growth restored by the addition of exogenous ATP, a metabolite depleted in Δ*tkl1* mutant strains. We show that ATP might act via the TOR signaling pathway, and TOR is likely a downstream target of activation for *TKL1*. *TKL1* is also involved in controlling the migration of appressorial nuclei into primary hyphae in host cells. When taken together, our results indicate transketolase has a novel role in mediating - via ATP and TOR signaling - an *in planta*-specific metabolic checkpoint that controls nuclear migration from appressoria into primary hyphae, prevents mitotic delay in early IH and promotes biotrophic growth. This work thus provides new information about the metabolic strategies employed by *M. oryzae* to enable rice cell colonization.

## Author Summary

The blast fungus *Magnaporthe oryzae* destroys rice and wheat harvests and could compromise global food security. Following penetration into the rice cell, *M. oryzae* elaborates bulbous invasive hyphae that grow in living rice cells for most of the infection cycle without causing disease symptoms. Little is known about the physiological processes governing this important biotrophic stage of fungal growth. Here, we used gene functional analysis to show how the primary metabolic enzyme transketolase is essential for hyphal growth in rice cells. Loss of transketolase did not affect the ability of the fungus to gain entry into rice cells, but invasive hyphal growth was curtailed in transketolase null mutants. Biotrophic growth was restored in transketolase mutants by the addition of exogenous ATP. We conclude that *M. oryzae* metabolism is dedicated to metabolizing glucose through transketolase *in planta* in order to provide ATP as a trigger for biotrophic growth and infection. This work is significant because it reveals important—but previously unknown—metabolic strategies employed by *M. oryzae* to facilitate rice infection. These strategies might be open to abrogation by chemical or biological means and are likely relevant to other rapidly proliferating intracellular pathogens.

## Introduction

The rice blast fungus *Magnaporthe oryzae* causes the most serious disease of cultivated rice [1], [2] and is a significant challenge to global food security [3], [4]. Infection involves the elaboration of a specialized structure, the appressorium, from a germinating three-celled conidium (spore) on the surface of the leaf [1], [5], [6]. Each cell of the conidium contains a nucleus, the most apical of which migrates into the germ tube during appressorium development where it undergoes closed mitosis [7]. One of the resulting daughter nuclei returns to the conidium to be degraded during autophagic cell-death [8]; the other enters the incipient appressorium [7] to become the source of *M. oryzae* genetic material during host infection. Disrupting autophagy or blocking the cell cycle at three checkpoints (S-phase, mitosis and exit from mitosis) abolishes infection [8], [9]. At maturation, enormous turgor is generated in the appressorium through the accumulation of glycerol and the ingress of water that becomes trapped due to a layer of melanin deposited on the wall of the appressorium [10], [11]. This hydrostatic pressure acts on a penetration peg [5], [6], forcing it through the leaf cuticle. In the cell lumen, the penetration peg becomes a thin primary hypha that invaginates the host plasma membrane before differentiating into bulbous invasive hyphae (IH) that are sealed in the plant-derived extra-invasive hyphal membrane (EIHM) compartment [12]. IH moves into adjacent cells by crossing the cell wall at plasmodesmata, and the biotrophic invasion process is repeated in successive rice cells [12]. After four to five days of biotrophic growth in susceptible cultivars, host cells die and *M. oryzae* enters its necrotrophic growth phase, causing spreading necrotic lesions on the leaf surface from which conidia are produced on aerial hyphae [1].

During appressorium development, lipid and glycogen stores in the spore are mobilized and transported to the appressorium in a manner dependent on the MAP kinase- and cAMP-dependent protein kinase A-signaling pathways [13], [14]. Following lipolysis in the incipient appressorium [15], a large body of evidence indicates that organelles and biochemical pathways that metabolize fatty acids, such as mitochondrial and peroxisomal β-oxidation and the glyoxylate cycle, are essential for the development of infection-competent appressoria [15]–[20]. Thus, fatty acid β-oxidation and the distribution of the resulting acetyl-CoA subunits into cellular processes such as cell wall biosynthesis [17] and pyruvate formation [19] are dominant biochemical pathways during appressorial development.

In contrast to appressorial function (which requires lipid catabolism through β-oxidation and the glyoxylate cycle), post-penetrative host infection is dependent on glucose 6-phosphate (G6P) sensing by trehalose-6-phosphate synthase 1 (Tps1) and the concomitant production of NADPH through the pentose phosphate pathway [2], [21]–[24]. The binding of G6P by Tps1 leads, via an NADPH-dependent signaling pathway, to the induced expression of genes encoding NADPH-requiring enzymes [22], [24] and the repression of genes required for utilizing alternative sources of carbon [23]. At least two NADPH-requiring processes under Tps1 control, the glutathione and thioredoxin antioxidation systems, are glucose-responsive and required for biotrophic growth in rice cells [24]. Taken together, we have proposed that these observations are consistent with the occurrence of a metabolic shift from lipid metabolism by *M. oryzae* on the host surface to glucose metabolism in the host cell [25]. This metabolic reprogramming hypothesis is supported by the observations that the glyoxylate enzyme isocitrate lyase is required for appressorium function but is not required for growth *in planta* [16]. However, which glucose metabolizing pathways are active during *M. oryzae* growth *in planta*, and whether glucose metabolism contributes to appressorial development, is not known.

Here, we sought to bolster our understanding of the metabolic strategies governing *M. oryzae in planta* growth by focusing on how glucose-metabolizing pathways contribute to rice blast infection. We used gene functional analysis and live-cell imaging to show how glucose-metabolizing pathways are not required for appressorium formation and function. Instead, glucose metabolism via transketolase (encoded by *TKL1*) is essential for the post-invasive colonization of rice cells by *M. oryzae*. The loss of transketolase function resulted in Δ*tkl1* mutant strains that could form appressoria, penetrate host cells and elaborate IH, but were attenuated for growth and cell cycle progression *in planta*. Δ*tkl1* strains were depleted for ATP, and IH growth was restored *in planta* following the treatment of Δ*tkl1* strains with exogenous ATP, suggesting ATP acts downstream of transketolase to signal growth [26], [27]. Moreover, evidence is provided to suggest ATP acts on the TOR signaling pathway and together our data indicate a functional connection between *TKL1*, ATP and the TOR signaling pathway. These results are consistent with a novel role for transketolase in mediating an *in planta*-specific metabolic checkpoint that regulates the cell cycle, via TOR activation, in order to permit rice cell colonization by *M. oryzae*. This work thus gives fresh insights into the metabolic demands of biotrophy and points to novel signaling roles for transketolase that will be important for other areas of biology.

## Results

### Early glycolysis, late gluconeogenesis and the pentose phosphate pathway are not required for appressorium formation

**Figure 1:**
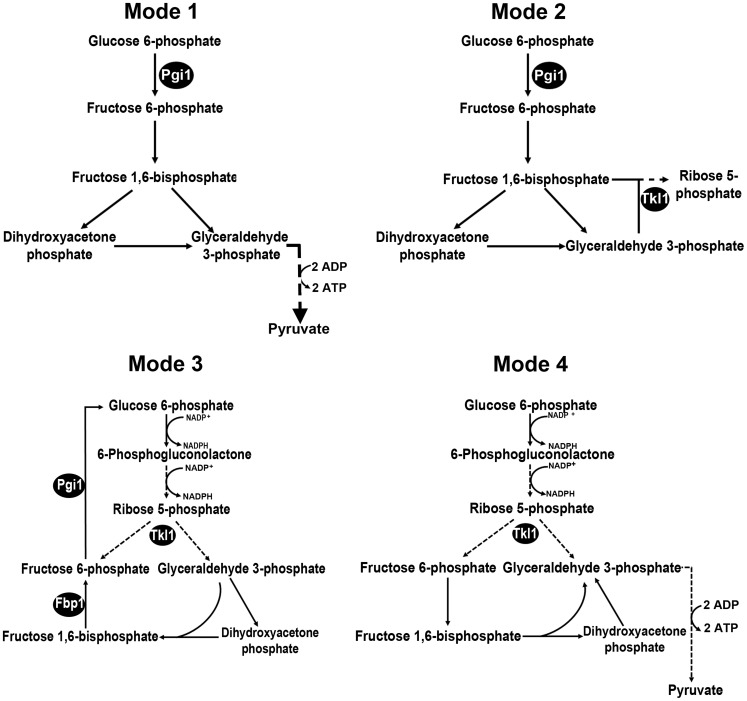
Potential metabolic destinations for G6P. Following Berg and colleagues, [28], we considered four scenarios for the fate of glucose 6-phosphate (G6P) during infection-related processes. **Mode 1**: More ATP than NADPH is required. G6P enters glycolysis through its conversion to fructose 6-phosphate by phosphoglucose isomerase 1 (Pgi1). **Mode 2**: *M. oryzae* cells require more ribose 5-phosphate for biosynthetic purposes than NADPH. Fructose 6-phosphate and glyceraldehyde 3-phosphate in the glycolytic pathway are converted to ribose 5-phosphate in the non-oxidative pentose phosphate pathway (PPP) by the action of transaldolase and transketolase (Tkl1). **Mode 3**: more NADPH than ribose 5-phosphate is required. G6P fuels NADPH production by G6P dehydrogenase (G6PDH) in the oxidative PPP and is resynthesized in the gluconeogenic pathway by fructose 1,6-bisphosphatase (Fbp1) and Pgi1. G6P is completely oxidized to CO_2_. **Mode 4**: NADPH and ATP are required. G6P fluxes through the PPP and into glycolysis via Tkl1 resulting in the production of NADPH and ATP. G6P is converted into pyruvate. Dashed lines indicate steps that have been omitted for clarity. Products of genes functionally characterized in the text are indicated by black spheres.

**Figure 2:**
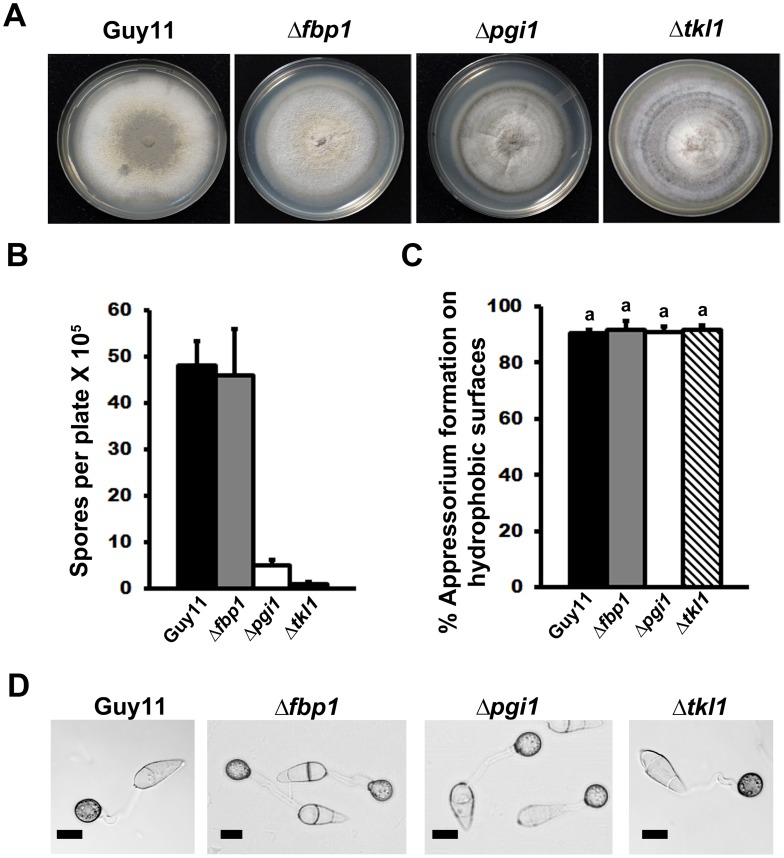
Dissection of G6P-utilizing pathways by gene functional analysis. (**A**) Radial growth on complete media (CM) was not impaired in mutant strains carrying gene deletions in *FBP1*, *PGI1* or *TKL1*. Guy11 is the wild type isolate used in this study. (**B**) Sporulation was impaired in strains lacking functional *PGI1* and *TKL1* genes, but not *FBP1*, during growth on CM. Results are the mean of three or more independent measurements. Error bars denote SD. Bars with the same letters are not significantly different (*Student's t-test* p≤0.05). (**C**) Spores of Guy11, Δ*pgi1*, Δ*fbp1* and Δ*tkl1* were applied to artificial hydrophobic surfaces. At 24 hr post inoculation (hpi), the rate of appressorial formation by either gene deletion strain was not significantly different to Guy11. Error bars are SD. Bars with the same letter are not significantly different (*Student's t-test* p≤0.05). (**D**) Loss of *PGI1*, *FBP1* or *TKL1* did not impair the formation of melanized appressoria on artificial hydrophobic surfaces by 24 hpi. Scale bar is 10 µm.

To develop a more robust understanding of the biochemical processes underlying plant infection by *M. oryzae*, we first sought to determine what glucose-metabolizing pathways might be important for rice infection. Following Berg and associates, [28], we considered four metabolic destinations for G6P in *M. oryzae*: into glycolysis via the action of phosphoglucose isomerase 1 (Pgi1) if more ATP than NADPH or ribose 5-phosphate is needed (Mode 1 in Figure 1); into early glycolysis and the non-oxidative pentose phosphate pathway if ribose 5-phosphate production for nucleotide biosynthetic purposes is predominant (Mode 2 in Figure 1); into the pentose phosphate pathway (PPP) and recycling via late gluconeogenesis until G6P is fully oxidized to CO_2_ if NADPH production is paramount (Mode 3 in Figure 1); into the PPP and glycolysis to form pyruvate if NADPH and ATP production is required (Mode 4 in Figure 1). Searching the *M. oryzae* genome [29], we found single orthologues of *FBP1* (MGG_08895) encoding fructose 1,6-bisphosphatase in the gluconeogenic pathway, *PGI1* (MGG_12822) involved in both glycolysis and gluconeogenesis, and *TKL1* (MGG_02471) encoding transketolase, a non-oxidative PPP enzyme linking the PPP and glycolysis. Using our high-throughput, PCR-based split marker method for gene deletion [22], we replaced part of the coding regions of *FBP1* and *PGI1* with the *ILV1* gene conferring sulphonyl urea resistance, and the coding region of *TKL1* with the *Bar* gene conferring bialaphos resistance. Figure 2A shows that the resulting three mutant strains - Δ*fbp1*, Δ*pgi1* and Δ*tkl1*- grew without impediment on glucose-rich complete media (CM) compared to wild type Guy11 strains. Despite these similarities in growth, Figure 2B shows that both *PGI1* and *TKL1*, but not *FBP1*, contributed significantly (*Student's t-test* p≤0.05) to sporulation rates on CM media. This indicates nucleotide biosynthesis via Mode 2 (Figure 1) might be important for sporulation, perhaps due to the nucleotide demands of DNA replication during spore production.

Interestingly, all the mutant strains in this study formed melanized appressoria on hydrophobic surfaces (Figure 2C and 2D) at rates that were indistinguishable from Guy11 (Figure 2C, *Student's t-test* p>0.05). This indicates that the glucose utilization pathways shown in Figure 1 are not required for appressorium formation and is consistent with previous work emphasizing the requirement for lipid metabolism and the glyoxylate cycle, rather than sugar metabolism, during appressorium formation [15]–[20]. Taken together, G6P metabolism via Mode 2 contributes to sporulation, but neither Mode described in Figure 1 is necessary for appressorial formation.

### Transketolase is essential for invasive hyphal growth in host rice cells

**Figure 3:**
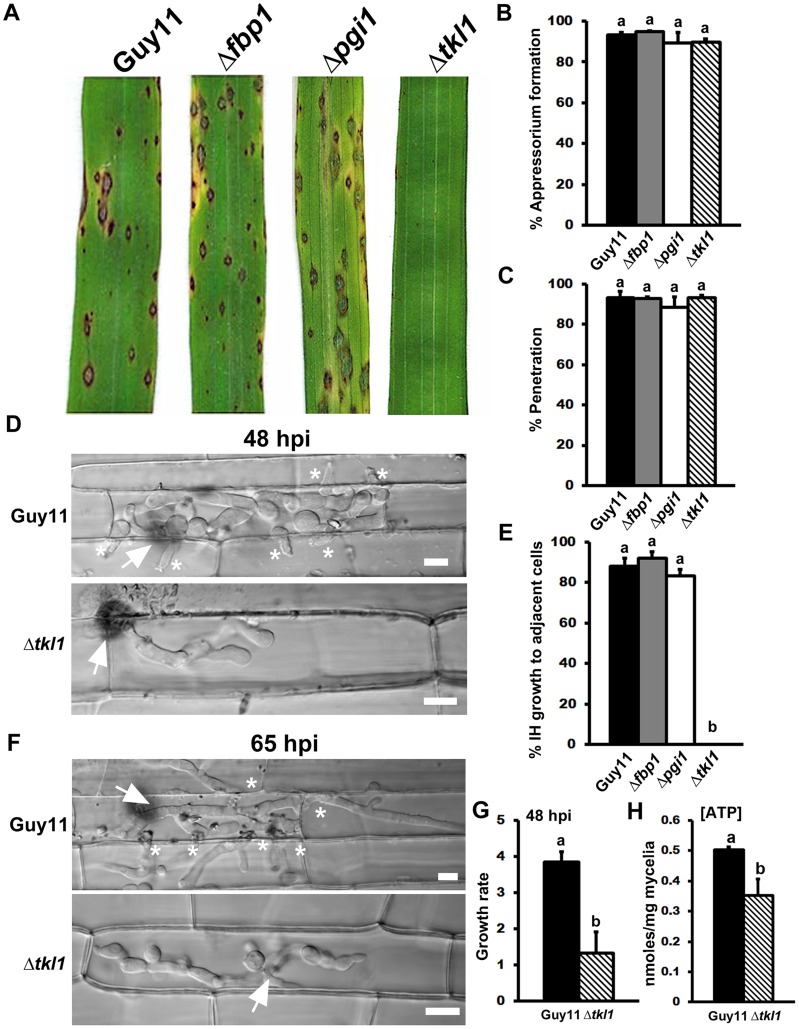
The transketolase-encoding gene *TKL1* is required for *in planta* growth. (**A**) *TKL1*, but not *PGI1* or *FBP1*, is essential for pathogenicity. Spore suspensions of each strain were applied to susceptible CO-39 plants at a rate of 1×10^5^ spores per mL. (**B**) Despite being non-pathogenic, the rate of appressorial formation by Δ*tkl1* strains was not significantly different to Guy11 on rice leaf surfaces at 24 hpi and (**C**) the rate of rice leaf penetration by Δ*tkl1* strains, determined at 30 hpi, was equivalent to Guy11. Values are the mean of three independent replicates. Error bars denote SD. Bars with the same letters are not significantly different (*Student's t-test* p≤0.05). (**D**) Live-cell imaging at 48 hpi of Guy11 and Δ*tkl1* strains infecting susceptible CO-39 rice leaf sheaths showed Δ*tkl1* was impaired in IH growth compared to Guy11. Scale bar is 5 µm. Arrows indicate appressoria on the surface of the leaf corresponding to the point of penetration. Asterisks indicate IH movement from primary infected cells to adjacent cells. (**E**) IH movement to cells adjacent to the point of penetration was impaired in Δ*tkl1* strains compared to Guy11, Δ*pgi* and Δ*fbp1* strains. Values are the percentage of IH in primary infected cells that have spread to neighboring cells at 48 hpi and represent the mean of three independent replicates. Error bars denote SD. Bars with the same letters are not significantly different (*Student's t-test* p≤0.05). (**F**) Live-cell imaging at 65 hpi of Guy11 and Δ*tkl1* strains infecting susceptible CO-39 rice leaf sheaths showed Δ*tkl1* was impaired in IH growth compared to Guy11. Scale bar is 5 µm. Arrows indicate appressoria on the surface of the leaf corresponding to the point of penetration. Asterisks indicate IH movement from primary infected cells to adjacent cells. (**G**) The *in planta* growth rate of Δ*tkl1* IH, determined at 48 hpi, was significantly reduced (*Student's t-test* p≤0.05) compared to Guy11. The growth rate of IH was measured by using the 1–4 scale described in [59]. Values are the mean of three independent replicates. Error bars denote SD. Bars with the same letters are not significantly different (*Student's t-test* p≤0.05). (**H**) Intracellular ATP levels were reduced in mycelia of Δ*tkl1* strains compared to Guy11 following growth in minimal media (MM). [ATP] was determined by LC-MS/MS, as described in Materials and Methods. Metabolite extractions were performed in triplicate for each strain. Error bars denote SD. Bars with the same letters are not significantly different (*Student's t-test* p≤0.05).

Although all the mutant strains were able to form appressoria on artificial hydrophobic surfaces, Figure 3A shows that when spores were applied to whole plants, only the Δ*tkl1* mutant strain was unable to cause disease. *TKL1* is thus revealed here as an essential and previously unknown determinant of pathogenicity by the rice blast fungus.

The inability of Δ*tkl1* strains to infect rice leaves was not due to defective appressorium formation or function on host surfaces because, like Δ*pgi* and Δ*fbp1* mutant strains, Δ*tkl1* formed appressoria on rice leaf surfaces at the same rates as Guy11 (Figure 3B; *Student's t-test* p = 0.64). Also, the rates of cuticle penetration on detached rice leaf sheaths were not significantly different (*Student's t-test* p = 0.37) for Guy11 and any of the mutant strains generated for this study, including Δ*tkl1* (Figure 3C).

If appressorium formation and function did not depend on a functional Tkl1 enzyme, how, then, does *TKL1* contribute to pathogenicity? To address this question, we continued to characterize the role of the*TKL1* gene in pathogenicity using live-cell imaging of detached rice leaf sheaths colonized with either Δ*tkl1* or Guy11 strains. At 48 hr post inoculation (hpi), Δ*tkl1* mutant strains were found to elaborate bulbous IH from primary hyphae, but IH growth was severely restricted to the first infected cell compared to Guy11 (Figure 3D). At 48 hpi, more than 80% of Guy11, Δ*pgi* and Δ*fbp1* primary infection sites had resulted in IH spreading to adjacent cells, but IH movement to adjacent cells by Δ*tkl1* mutant strains was not observed (Figure 3E). Δ*tkl1* strains had also not spread to adjacent cells by 65 hpi (Figure 3F). Growth rates in rice cells at 48 hpi was scored for 50 infected cells/strain (repeated in triplicate) using our 4 point scale (where 1 = IH length shorter than 10 µm with no branching; 2 = IH length is 10–20 µm with 0–2 branches; 3 = IH length is longer than 20 µm and/or with more than 2 branches within one cell; 4 = IH has spread to adjacent cells). The average growth rate score for Δ*tkl1* strains in rice cells at 48 hpi was significantly reduced compared to Guy11 (Figure 3G; *Student's t-test* p = 0.0078). Therefore, *TKL1* is not required for appressorium formation or function, or for IH elaboration, but is essential for strong biotrophic growth in rice cells and the movement of IH to neighboring cells.

### Glucose is metabolized via transketolase during rice infection

Figure 3A and 3E shows that early glycolytic and late gluconeogenic steps are dispensable for biotrophic growth in rice cells and disease development, whereas the non-oxidative PPP enzyme Tkl1 is essential. This indicates that G6P metabolism via Mode 4 (Figure 1) is likely the dominant pathway for glucose utilization by *M. oryzae* during host infection. Further experimental support for Mode 4—which would involve Tkl1 connecting the PPP and later glycolytic steps to yield NADPH and ATP from glucose [28]—is shown in Figure 3G where, following axenic growth in minimal media (MM), Δ*tkl1* mycelia was found by LC-MS/MS analysis to contain significantly less ATP than the mycelia of Guy11 strains (Figure 3H). Therefore, ATP production is disrupted in Δ*tkl1* strains.

### Δ*tkl1* mutant strains are downregulated for genes associated with translation initiation and protein biosynthesis *in planta*

**Figure 4:**
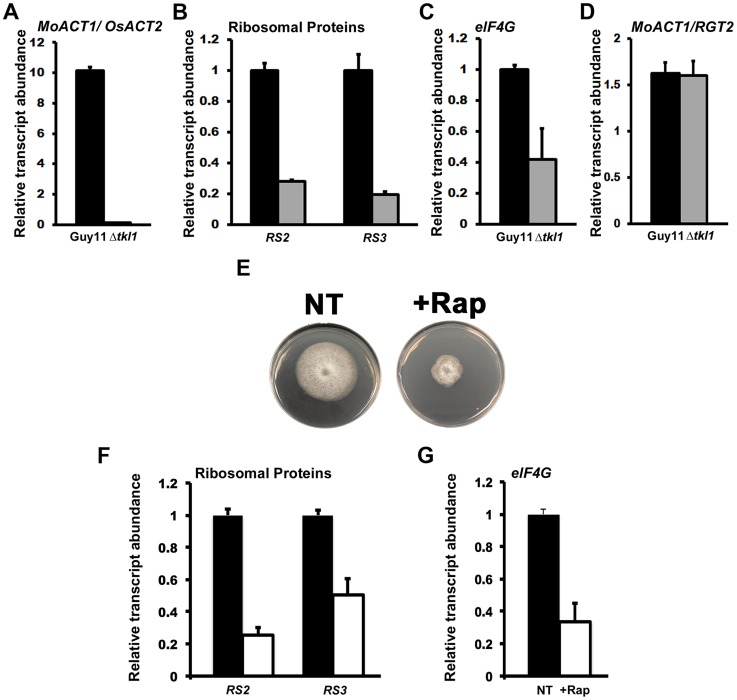
*In planta* gene expression in Δ*tkl1* strains suggests attenuated growth. (**A–D**) *In planta* gene expression was analyzed by qPCR using cDNAs generated from infected rice leaf sheaths at 48 hpi. Values are the mean of three independent replicates. Error bars are SD. Closed bars are Guy11 expression levels; open bars are Δ*tkl1* expression levels. (**A**) Guy11 was 10 fold more abundant than Δ*tkl1* strains in rice cells at 48 hpi, as determined from the expression of *MoACT1* normalized against rice actin (*OsACT2*) gene expression. Values are given as fold differences between each strain. Closed bar is the ratio of the mean normalized *MoACT1* Ct values for Guy11: mean normalized *MoACT1* Ct values for Δ*tkl1*; open bar is the ratio of the mean normalized *MoACT1* Ct values for Δ*tkl1*: mean normalized *MoACT1* Ct values for Guy11. (**B**) The expression of the *M. oryzae* ribosomal protein genes *RS2* and *RS3* was reduced *in planta* in Δ*tkl1* strains compared to Guy11 when normalized against *MoACT1* expression. Expression changes in Δ*tkl1* strains are shown relative to Guy11. (**C**) *eIF4G* expression was downregulated in Δ*tkl1* strains compared to Guy11 in planta following normalization against *MoACT1* expression. Expression changes are relative to Guy11. (**D**) Expression levels of *MoAct1*, relative to a second constitutively expressed gene *RGT2*, were not different between Guy11 and Δ*tkl1* strains *in planta* at 48 hpi. (**E**) The radial growth of Guy11 on minimal media (MM) containing the preferred carbon source glucose and the preferred nitrogen source ammonium was compromised when 50 nM (final concentration) of rapamycin, the specific TOR kinase inhibitor, was added to the media. + Rap = 50 nM rapamycin was added to the media. NT = no rapamycin treatment. (**F**) The gene expression levels of *RS2*, *RS3* and (**G**) *eIF4G* were reduced in Guy11 following growth in MM containing glucose and ammonium in the presence of 50 nM rapamycin. Values are the mean of three independent replicates. Error bars are SD. Closed bars are gene expression levels following growth in media without rapamycin treatment; open bars are gene expression levels following growth in media with 50 nM rapamycin treatment. Expression levels were normalized against *TUB2* gene expression and are given relative to Guy11 without rapamycin treatment.

We next sought to determine the molecular basis for the observed attenuated growth of Δ*tkl1* mutant strains *in planta* (Figure 3). First, we wished to ascertain if Δ*tkl1* mutants were senescent or killed in host cells after a short period of initial IH growth. We used quantitative real time PCR (qPCR) to measure *M. oryzae* actin (*MoACT1*) gene expression in Guy11 and Δ*tkl1* mutant strains, normalized against rice actin (*OsACT2*) gene expression, during the infection of rice epidermal cells at 48 hpi. The ratio of *MoACT1* to *OsACT2* gene expression was significantly higher in rice cells infected with Guy11 than Δ*tkl1* (Figure 4A). This was to be expected considering the greater growth rate of Guy11 in rice cells (Figure 3G). Nonetheless, *MoACT1* gene expression was detectable in rice cells infected with Δ*tkl1* mutant strains. CT values for *MoACT1* expression, before rice actin normalization, were 28.5±0.2 for rice cells infected with Δ*tkl1* mutants compared to 23.7±0.1 for those infected with Guy11. These results indicate Δ*tkl1* mutant strains were alive in rice cells at 48 hpi and undertaking some gene transcription despite the reduced growth rate of these strains (Figure 3G).

Because Δ*tkl1* mutant strains were alive in rice cells, we next asked what molecular mechanism might account for their attenuated growth *in planta*. Quiescence is associated with arrested growth and has been described as either an extended G_1_ stage or a distinct G_0_ state [26], [27]. Severely reduced growth states such as those exhibited by Δ*tkl1* strains also exhibit some features of quiescence [26], [27], [30] and it is not known if quiescence is a distinct state from slow growth [31]. Cellular quiescence occurs in yeast and mammals via the inactivation of the TOR signaling pathway in response to nutrient depletion. Conversely, the activated TOR signaling pathway promotes growth under favorable nutrient conditions. TOR inactivation results in, amongst other outcomes, cell cycle arrest [32] and translation suppression [33], [34]. Translation suppression occurs at the level of ribosome biogenesis, including TOR-dependent downregulation of ribosomal gene expression [35], [34]. To determine if the TOR signaling pathway might play a role in the poor growth of Δ*tkl1* strains in rice cells, we first examined the expression of two ribosomal genes, *RS2* and *RS3* (encoding ribosomal protein S2-like and 40S ribosomal protein S3, respectively), and the translation initiation factor *eIF4G*, in Guy11 and Δ*tkl1* mutant strains *in planta* at 48 hpi. *eIF4G* expression is reduced when mammalian mTOR is inhibited in an immortalized breast epithelial cell, and *eIF4G* depletion results in impaired cell proliferation and increased autophagy [36]. Compared to Guy11, and following normalization against *MoACT1*, both ribosomal protein genes (Figure 4B) and *eIF4G* (Figure 4C) were downregulated in Δ*tkl1* mutant strains *in planta*.

To ensure that the observed downregulation of gene expression in Δ*tkl1* mutants *in planta* was not attributable to a general decrease in *MoACT1* expression in Δ*tkl1* strains compared to Guy11, we analyzed the expression of a putative glucose transporter encoded by *RGT2*, previously shown to be highly and constitutively expressed during rice infection [37]. Figure 4D shows that the ratio of *MoACT1* gene expression to *RGT2* gene expression, *in planta*, is the same for both Guy11 and Δ*tkl1*, indicating *MoACT1* gene expression was not reduced in Δ*tkl1* strains compared to Guy11.

We next sought to determine if *RS2*, *RS3* and *eIF4G* gene expression was under TOR signaling control in *M. oryzae*. We studied the expression levels of these genes under optimal growth conditions (ie. minimal media containing the preferred carbon and nitrogen sources glucose and ammonium, respectively [2], [23]), and following growth on the same media containing the specific TOR kinase inhibitor rapamycin [33], [35]. Figure 4E shows that Guy11 grew poorly on preferred media when 50 nM rapamycin was added, confirming that optimal growth requires an activated TOR signaling pathway. Figure 4F and 4G shows, consistent with observations in yeast and mammalian cells, that poor growth in media containing rapamycin was associated with *RS2*, *RS3* and *eIF4G* downregulation. Taken together, Figure 4 suggests Δ*tkl1* mutant strains might be downregulated - in a TOR-dependent manner - for translation initiation and protein biosynthesis during growth in rice cells.

### Mitosis is delayed in Δ*tkl1* mutant strains *in planta*

**Figure 5:**
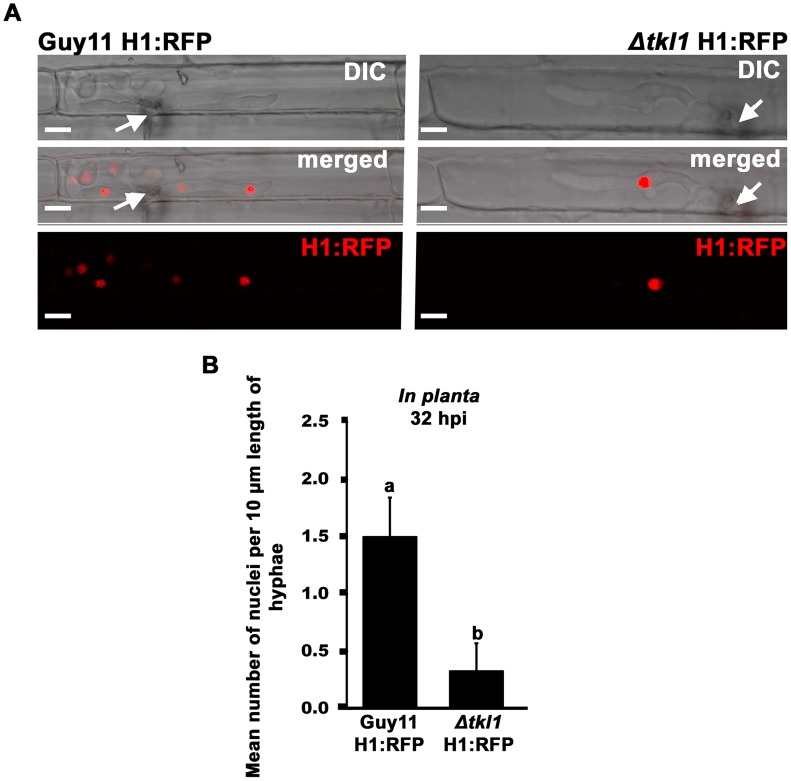
Nuclear division is attenuated in Δ*tkl1* mutant strains *in planta*. (**A**) Live-cell imaging at 32 hpi of Guy11 H1:RFP and Δ*tkl1* H1:RFP strains infecting susceptible CO-39 rice leaf sheaths showed Δ*tkl1* H1:RFP strains were impaired in nuclear proliferation in epidermal cells compared to Guy11 H1:RFP. Arrows indicate point of penetration. Scale bar is 5 µm. (**B**) The mean number of nuclei in 10 µm lengths of IH was calculated, using ImageJ, for each strain at 32 hpi, indicating Δ*tkl1* H1:RFP strains were delayed in mitosis. Values are the mean of at least six independent replications. Error bars denote SD. Bars with the same letters are not significantly different (*Student's t-test* p≤0.05).

In response to nutrient deprivation, TOR inactivation can arrest yeast cells in G_1_ to induce quiescence [33]. In addition, TOR inactivation prolongs the G_2_/M transition in yeast [33]. Thus, cell growth and cell cycle progression are linked by common signaling pathways, including TOR [33]. In order to continue investigating the role of *TKL1* during *in planta* growth, we next turned our attention to the cell nucleus. We performed live-cell imaging of *M. oryzae* IH using a Guy11 strain expressing a histone H1 protein fused to the tdTomato variant [38] of red fluorescent protein (H1:RFP) - described in [9] - and the same strain lacking a functional copy of *TKL1* due to homologous recombination with the *ILV1* gene conferring sulphonyl urea resistance (Δ*tkl1* H1:RFP). The individual nuclei of these strains were visualized by epifluorescence microscopy, and Figure 5A shows that at 32 hpi, when both strains had elaborated IH in rice cells and growth differences between the two strains were not as pronounced as at later timepoints, the IH of Guy11 H1:RFP contained significantly more (*Student's t-test* p≤0.05) nuclei, per infected rice cell, than the IH of Δ*tkl1* H1:RFP strains (quantified below). We also quantified the rate of mitosis in both strains at 32 hpi by measuring the number of nuclei per unit length of mycelia. Figure 5B shows that 10 µm lengths of Δ*tkl1* H1:RFP IH carried significantly less nuclei (*Student's t-test* p≤0.05) than 10 µm lengths of Guy11 H1:RFP IH. Thus, attenuated *in planta* growth by Δ*tkl1* strains is accompanied by delayed (but not arrested) mitotic progression. Interestingly, no significant differences in nuclei number (*Student's t-test* p>0.05) were observed in Δ*tkl1* H1:RFP vegetative hyphae compared to Guy11 H1:RFP following the growth of both strains in glucose rich CM (Figure S1A and B) or in defined glucose minimal media (GMM) with varying concentrations of glucose (Figure S2). This is consistent with Figure 2A that shows*TKL1* is not required for optimal radial growth on CM.

Together with the transcript data presented in Figure 4, and the physiological data in Figure 3, the results presented in Figure 5 support the notion that a functional *TKL1* gene is required for cell cycle progression and growth during the biotrophic colonization of host rice cells.

### Mitotic delay in Δ*tkl1* mutant strains is obviated by treatment with ATP

Why does loss of *TKL1* result in mitotic delay and growth attenuation? We speculated that, considering the Tkl1 enzyme is involved in glucose metabolism, the lack of a metabolite(s) or metabolic pathway(s) downstream of Tkl1 might impact, directly or indirectly, cell cycle progression. From Figure 3H we knew that Δ*tkl1* strains were significantly reduced in ATP production compared to Guy11, such as would be predicted from perturbations to Mode 4 (Figure 1). In the absence of evidence for other metabolic changes, we hypothesized, then, that treatment of Δ*tkl1* mutant strains with exogenous ATP might remediate IH growth and affect mitotic rates upon host infection.

**Figure 6:**
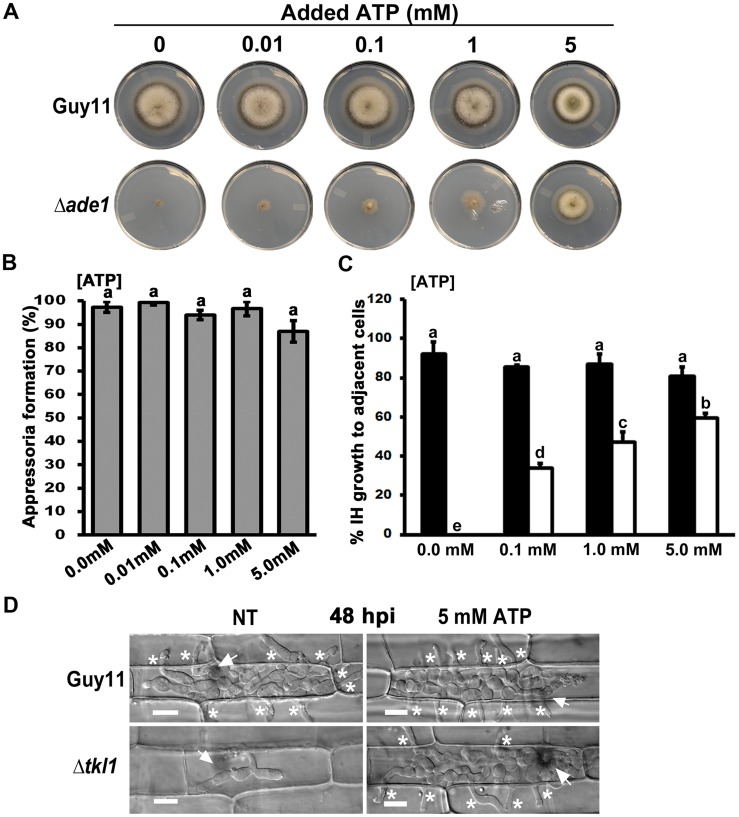
Exogenous ATP promotes biotrophic growth and cell-to-cell movement by Δ*tkl1* mutant strains. (**A**) Exogenous ATP can remediate growth of the adenine requiring mutant Δ*ade1* on MM at the final concentrations indicated. Plates were imaged after 10 days of growth. (**B**) Spores of Δ*tkl1* were treated with the indicated concentrations of ATP and applied to rice leaf sheaths. Appressorium formation rates were determined at 24 hpi by counting the number of appressoria that had elaborated from 50 spores/leaf sheath for each treatment. Values are the mean of three independent replicates. Error bars denote SD. Bars with the same letters are not significantly different (*Student's t-test* p≤0.05). (**C**) Spores of Guy11 (closed bar) or Δ*tkl1* (open bar) were treated with ATP at the indicated final concentrations before applying to detached leaf surfaces. The rate of cell-to-cell movement was determined by counting how many of 50 primary infected cells had colonized adjacent cells by 48 hpi. Values are the mean of three independent replicates. Error bars denote SD. Bars with the same letters are not significantly different (*Student's t-test* p≤0.05). (**D**) Compared to untreated controls, the treatment of Δ*tkl1* spores with 5 mM ATP resulted in increased cell-to-cell movement in detached leaf sheaths at 48 hpi. NT = no treatment. Scale bar is 5 µm. Arrows indicate appressoria on the surface of the leaf corresponding to the point of penetration. Asterisks indicate IH movement from primary infected cells to adjacent cells.

To test this hypothesis, we first needed to determine if *M. oryzae* was capable of acquiring or metabolizing ATP from the external milieu. In a previous study, we had shown how an adenine-requiring mutant, Δ*ade1*, could not grow on minimal media unless supplemented with adenosine or adenine [37]. Figure 6A shows how this same Δ*ade1* mutant strain was remediated for growth on minimal media in the presence of ATP at a final concentration of 5 mM. This suggests that *M. oryzae* is capable of acquiring exogenous ATP directly from the media and, in adenine-requiring Δ*ade1* mutant strains, this remediates axenic growth by converting ATP to adenine through the purine salvage pathway [37]. In addition, because Δ*ade1* mutant strains can penetrate host cells but fail to establish infection, the growth tests in Figure 6A indicate plant sources of ATP, like adenine and adenosine [37], are not available to *M. oryzae* during growth in rice cells.

To determine whether exogenous ATP might interfere with the cAMP-signaling pathway controlling appressorium development [1], we added increasing amounts of ATP to Guy11 spores and applied them to leaf surfaces. No significant (*Student's t-test* p≤0.05) inhibition of appressorium formation was observed using final concentrations up to and including 5 mM ATP (Figure 6B). Δ*tkl1* spores treated with the same concentrations of ATP were then applied to detached leaf sheaths. Live-cell imaging showed cell-to-cell movement was improved in Δ*tkl1* mutant strains following treatment with ATP in a dose-dependent manner (Figure 6C and 6D).

**Figure 7:**
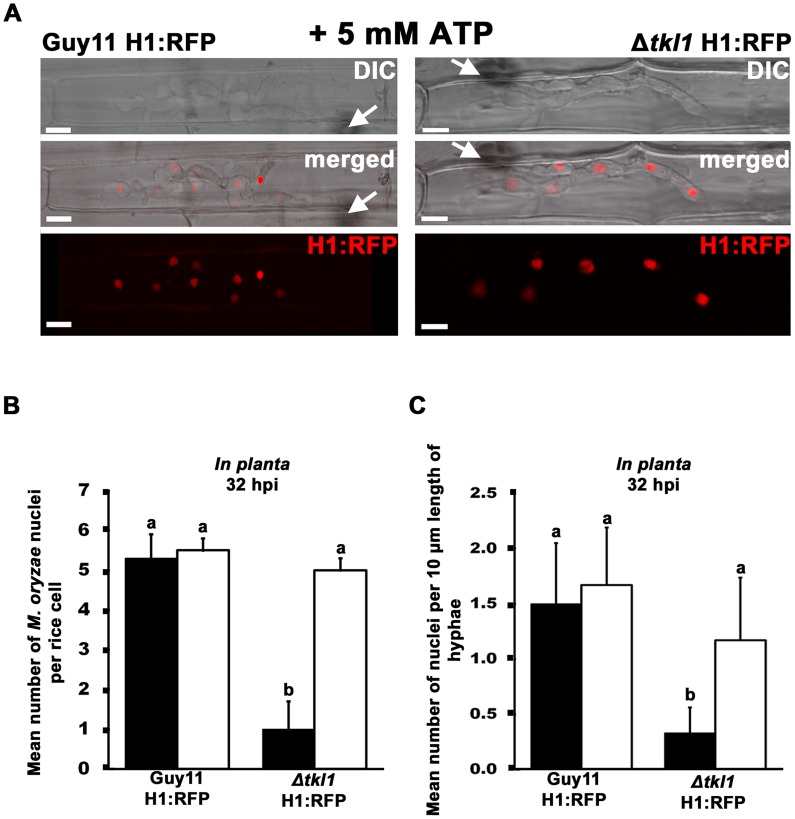
Mitotic delay is remediated *in planta* by treating Δ*tkl1* H1:RFP mutant strains with exogenous ATP. (**A**) Spores of Guy11 H1:RFP and Δ*tkl1* H1:RFP strains were treated with 5 mM ATP and applied to rice leaf sheaths. Live-cell imaging at 32 hpi shows ATP treated Δ*tkl1* H1:RFP strains were restored for nuclear proliferation in rice epidermal cells compared to Guy11 H1:RFP. Arrows indicate point of penetration. Scale bar is 5 µm. (**B**) Spores of Guy11 H1:RFP and Δ*tkl1* H1:RFP strains were either untreated (closed bar) or treated with 5 mM ATP (open bar) and applied to rice leaf sheaths. The mean total number of nuclei produced by Guy11 H1:RFP and Δ*tkl1* H1:RFP *in planta* at 32 hpi was calculated for each infected cell. Nuclei were counted in 50 infected cells per leaf sheath. Values are the mean of three independent replicates. Error bars denote SD. Bars with the same letters are not significantly different (*Student's t-test* p≤0.05). (**C**) The mean number of nuclei in 10 µm lengths of IH was calculated, using ImageJ, at 32 hpi for each strain. Closed bars are untreated controls, open bars are strains treated with 5 mM ATP. Values are the mean of at least six independent replications. Error bars denote SD. Bars with the same letters are not significantly different (*Student's t-test* p≤0.05).

We next added 5 mM ATP to spores of our RFP labeled histone H1 strains and applied them to detached rice leaf sheaths. Figure 7A and 7B shows how the addition of exogenous ATP increased the total number of nuclei in the IH of Δ*tkl1* H1:RFP, in rice cells at 32 hpi, to levels comparable to those observed in the IH of Guy11 H1:RFP. The rate of mitosis was also remediated in Δ*tkl1* H1:RFP strains by ATP treatment (Figure 7C). Similarly, at 48 hpi, the mitotic rate of untreated Δ*tkl1* H1:RFP was significantly less (*Student's t-test* p≤0.05) than Guy11 H1:RFP but was restored by ATP treatment (Figure S3A). However, at 65 hpi, there was no significant difference (*Student's t-test* p≤0.05) in the rates of mitosis between Δ*tkl1* H1:RFP and Guy11 H1:RFP strains in untreated samples (Figure S3B), suggesting mitotic delay is surmounted in Δ*tkl1* strains at later timepoints, perhaps in response to other metabolites.

The remediating effect of ATP on Δ*tkl1* H1:RFP IH nuclear proliferation at 32 hpi was also observed when Δ*tkl1* H1:RFP spores were treated with the related compound adenosine (possibly due to its conversion to ATP through the purine salvage pathway [37]), but not when treated with 0.4 mM of the unrelated ROS quencher diphehyleneiodonium chloride (data not shown). Thus, we conclude exogenous sources of ATP can stimulate cell cycle progression in Δ*tkl1* mutant strains.

### Δ*tkl1* mutant strains are not impaired in mitosis or autophagy during appressorium development

**Figure 8:**
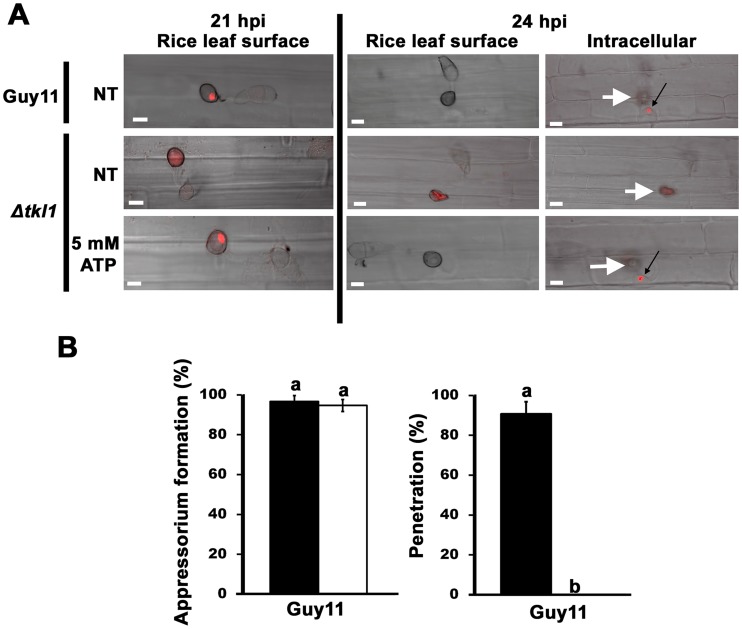
*TKL1* is required for the migration of appressorial nuclei into primary hyphae in host cells. (**A**) Spores of Guy11 H1:RFP and Δ*tkl1* H1:RFP strains were either not treated (NT) or treated with 5 mM ATP and applied to rice leaf sheaths. At 21 hpi, untreated Guy11 H1:RFP and Δ*tkl1* H1:RFP strains treated with 5 mM ATP displayed a single punctate nucleus in the appressorium which had migrated into primary hyphae by 24 hpi (black arrows). In contrast, untreated Δ*tkl1* H1:RFP strains lacked a punctate nuclear structure at 21 hpi, and no punctate nucleus was evident in primary hyphae by 24 hpi. All panels are merged DIC and fluorescence images. Scale bar is 10 µm. White arrows indicate appressoria on the surface of the leaf corresponding to the point of penetration. (**B**) Spores of Guy11 were either untreated (closed bars) or treated (open bars) with 5 mM AMP-PNP, a non-hydrolysable analogue of ATP. Appressorium formation on rice leaf sheaths was unaffected by AMP-PNP treatment, but penetration was abolished. For each treatment, appressorium formation rates were determined at 24 hpi by counting the number of appressoria that had elaborated from 50 spores/leaf sheath for each treatment. Penetration rates were determined at 24 hpi by counting the number of successful cuticle breaches made by 50 appressoria/leaf sheath. Values are the mean of three independent replicates. Error bars denote SD. Bars with the same letters are not significantly different (*Student's t-test* p≤0.05).

The preceding results demonstrated that following host penetration, nuclear division rates in the IH of Δ*tkl1* H1:RFP mutant strains were reduced compared to those of Guy11 H1:RFP, but this mitotic delay could be avoided by treatment with ATP prior to infection. However, untreated Δ*tkl1* mutant strains could still form infection-competent appressoria on the host surface (Figure 3B and C). This was interesting considering appressorial developmental involves, amongst other processes, cAMP signaling [1] and an active cell cycle [7], [9]. How can these observations be reconciled? To address this question, we applied spores of Guy11 H1:RFP and Δ*tkl1* H1:RFP strains to detached leaf surfaces and observed nuclei behaviour during appressorium development and early infection. Figure S4 shows that, up to 21 hpi, there was little difference in the behaviour of the nuclei of either strain, with each strain demonstrating three conidial nuclei at 13 hpi, and a punctate, appressorial nucleus at 18 hpi. Figure 8A and Figure S4 shows that by 21 hpi, no nuclei remained in the conidium of either strain, indicating that appressorial development and autophagic cell death of the conidium and its contents [8] had progressed normally in both strains. The timing of these events on rice cuticles is consistent with what has previously been demonstrated during appressorial formation on artificial hydrophobic surfaces [9]. However, at 21 hpi, whereas H1:RFP was still localized to a single, punctate appressorial nucleus in Guy11 H1:RFP strains, H1:RFP was diffused throughout the appressoria of Δ*tkl1* H1:RFP strains (Figure 8A and Figure S4). Differences were also seen at 24 hpi when, by this time, the single nucleus of each Guy11 H1:RFP appressorium had migrated from the leaf surface to primary hyphae in the host cell (Figure 8A), but no nuclear migration was evident for Δ*tkl1* H1:RFP (Figure 8A). However, Δ*tkl1* H1:RFP strains treated with ATP displayed a single, punctate appressorial nucleus at 21 hpi which had migrated into primary hyphae by 24 hpi (Figure 8A). These results suggest *TKL1* is not required for the cell cycle events that give rise to the single nucleus of the appressorium, or for autophagic cell death and degradation of the remaining nuclei in the conidium. Instead, the results presented here indicate *TKL1* acts after autophagy, via ATP, to maintain a punctate appressorial nucleus and control its migration into primary hyphae in the host cell.

Additional evidence that ATP acts after appressorial formation comes from the use of the non-hydrolysable analogue of ATP, adenosine 5′-adenylyl imidodiphosphate (AMP-PNP). Treating Guy11 spores with AMP-PNP did not prevent appressorium formation on detached leaf sheaths but did abolish appressorium penetration of host surfaces (Figure 8B). This could indicate AMP-PNP is not taken up from the environment until after the appressorium has formed, but this is not likely considering cAMP is readily taken up into the cell prior to appressorial development [25]. Rather, the results with AMP-PNP are consistent with *TKL1* and ATP being necessary for cuticle penetration and post-penetration development but not appressorial development and autophagy.

### The *M. oryzae* TOR signaling pathway is activated downstream of *TKL1* in response to ATP

The results presented above suggest *TKL1* temporally controls the migration of appressorial nuclei into primary hyphae, and is required for the subsequent establishment of IH biotrophic growth, using a mechanism that involves ATP. Because TOR inactivation slows growth and extends mitosis [33], [34], we considered that a functional relationship between *TKL1*, ATP and the TOR signaling pathway might exist to promote biotrophic growth *in planta*.

**Figure 9:**
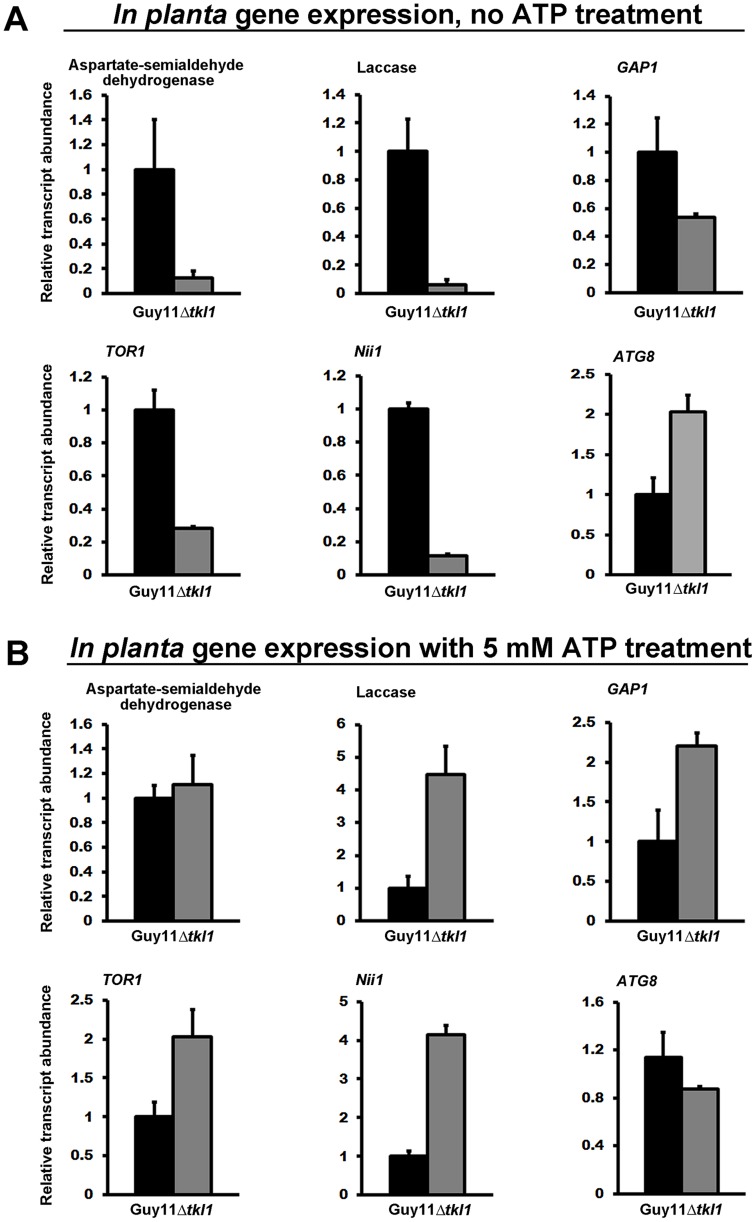
*TKL1* regulates the expression of TOR pathway read-out genes, via ATP, *in planta*. *M. oryzae in planta* gene expression was analyzed by qPCR using cDNAs generated from infected rice leaf sheaths at 48 hpi. Transcript abundance was normalized against *MoACT1* gene expression. Expression changes in Δ*tkl1* strains are shown relative to Guy11. Values are the mean of three independent replicates. Error bars are SD. Closed bars are Guy11 expression levels; open bars are Δ*tkl1* expression levels. (**A**) Without ATP treatment, genes under positive TOR pathway control were downregulated in Δ*tkl1* strains compared to Guy11. By contrast, the *ATG8* gene was upregulated in Δ*tkl1* strains compared to Guy11. (**B**) *In planta* Δ*tkl1* gene expression patterns were reversed when spores were treated with 5 mM ATP before applying to the host surface.

To tests this hypothesis and thus gain more mechanistic insights into the regulation of *M. oryzae* gene expression during biotrophic growth, we studied the transcript levels for a number of known TOR read-out genes [34], [35], [39] in Guy11 and Δ*tkl1* mutant strains *in planta* at 48 hpi. These included *M. oryzae* genes shown in a previous study [39], or known from yeasts studies [35], to be positively regulated by the activated TOR pathway, such as those involved in nitrogen uptake and utilization (*Nii1* encoding nitrate reductase, an aspartate semi-aldehyde dehygrogenase-encoding gene and *GAP1* encoding the general amino acid permease [39]), a laccase putatively involved in cell wall modifications [39], and the single *M. oryzae* TOR-encoding gene *TOR1* [35]. We also studied the expression of the *ATG8* gene involved in autophagy [8], a process upregulated when TOR signaling is inactivated [34]. Figure 9A shows that all TOR readout genes were downregulated in expression in Δ*tkl1* strains, *in planta*, compared to Guy11 except *ATG8*, which was more highly expressed in Δ*tkl1* strains. These expression patterns are consistent with reduced TOR activity in Δ*tkl1* strains compared to Guy11 during *in planta* biotrophic growth at 48 hpi. Gene expression patterns were restored in Δ*tkl1* mutant strains when the infection was repeated using Guy11 and Δ*tkl1* spores treated with 5 mM ATP (Figure 9B). These results provide evidence that *TKL1* activates the TOR signaling pathway via ATP (or an ATP derivative) in order to regulate gene expression *in planta*, promote biotrophic growth and prevent mitotic delay.

**Figure 10:**
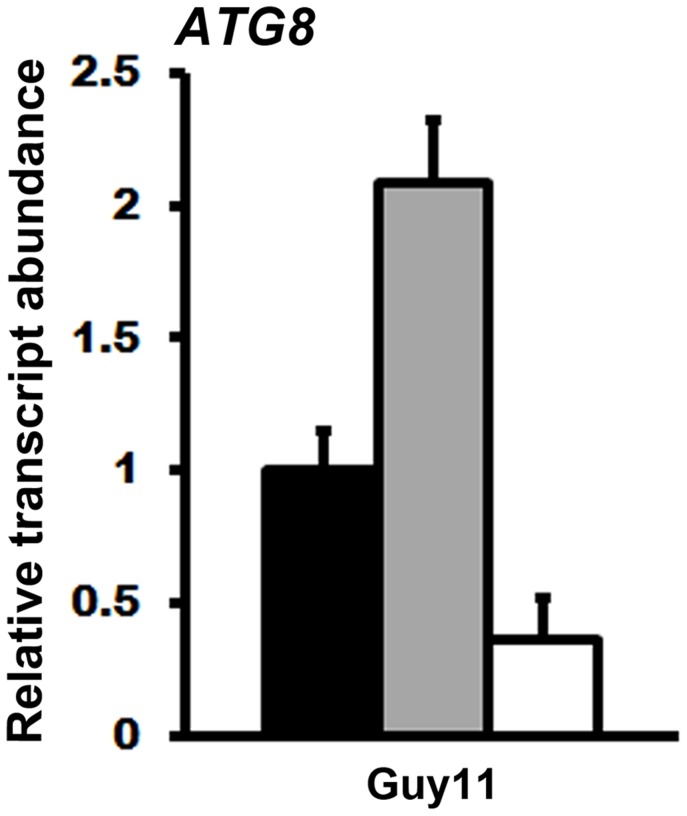
Exogenous ATP activates the TOR signaling pathway. cDNA for qPCR analysis of *ATG8* expression was obtained from the mycelia of Guy11 grown for 16 hr in liquid MM with 1% glucose and 10 mM ammonium (GMM) (closed bar), GMM with 50 nM rapamycin (grey bar), and GMM containing both 50 nM rapamycin and 5 mM ATP (open bar). Transcript abundance was normalized against *TUB2* expression and gene expression changes are shown relative to *ATG8* expression levels in GMM. Values are the mean of three independent replicates. Error bars are SD.

Further evidence for a connection between ATP and the TOR signaling pathway in *M. oryzae* is shown in Figure 10. Compared to axenic growth in liquid minimal media with glucose and ammonium (preferred carbon and nitrogen sources for *M. oryzae* [2], [23]), growth on the same media with 50 nM rapamycin [33], [35] resulted in elevated expression of the autophagy gene *ATG8*. This observation is consistent with studies in yeast, which showed that rapamycin inhibits the TOR signaling pathway and activates autophagy [33]–[35]. However, rapamycin induction of *ATG8* expression in *M. oryzae* was not observed when exogenous ATP was also present (Figure 10). Thus in *M. oryzae*, at least under some growth conditions, exogenous ATP can positively influence the TOR signaling pathway and override negative-acting signals such as those deriving from rapamycin treatment.

In summary, the results presented above suggest that *TKL1* and ATP influence the expression of at least some known TOR read-out genes during biotrophic growth. How ATP affects the TOR signalling pathway, and the implications for biotrophy, require future elucidation.

## Discussion

*M. oryzae* is a serious threat to world rice harvests and spends most of its infection cycle as a symptomless biotroph growing cell-to-cell in rice leaves before the onset of necrosis. Compared to appressorial development on the host surface, little is known about the metabolic and physiological demands of the fungus during *in planta* growth [25]. Improving our basic understanding of the biology of biotrophy might contribute to devising effective, long-term resistance strategies using novel chemical or biological interventions. Here, we aimed to add to our knowledge of *M. oryzae* by describing how some glucose-metabolizing pathways contribute to rice blast disease. In doing so, we revealed that *TKL1*, encoding transketolase, is essential for pathogenicity and controls biotrophic growth in rice cells via a mechanism involving ATP, TOR and the regulation of cell cycle progression.

This work started with the hypothesis that *M. oryzae* undergoes metabolic reprogramming during infection, switching from lipid metabolism on the surface of the leaf during appressorium formation to glucose metabolism in host cells. On the leaf surface, β-oxidation and the glyoxylate cycle are required for developing infection-competent appressoria [16], [17], but at least the glyoxylate enzyme isocitrate lyase is not required for growth *in planta* [16]. In contrast, our genetic dissections of glucose metabolism in *M. oryzae* show that early glycolysis, late gluconeogenesis and the PPP are not required for appressorium formation or function on the rice leaf surface (Figure 3B and C). Rather, glucose utilization through transketolase (but not early glycolysis or late gluconeogenesis) is essential for growth in host cells (Figure 3A and D). Glucose utilization through transketolase likely occurs in the direction of glycolysis because ATP levels are reduced in the mycelia of Δ*tkl1* strains compared to Guy11 (Figure 3H). Together, the genetic and biochemical evidence point to transketolase being important in connecting the PPP with glycolysis during *in planta* growth (Mode 4, Figure 1).

The critical post-penetration, *in planta*-specific role of *TKL1* indicates that once in the host cell, *M. oryzae* metabolism might be dedicated to NADPH and ATP production from glucose. NADPH is likely important, amongst other processes, for recycling antioxidation systems [40], [41] and maintaining redox balance during biotrophic growth [24]; ATP is likely required for meeting the energetic demands of the fungus *in planta*. A major and novel finding of this work is that ATP produced via Tkl1 can also act as a signal, likely upstream of the TOR pathway, to promote cell cycle progression and trigger the biotrophic growth of *M. oryzae in planta*. Delayed mitotic progression was reversed by the addition of exogenous ATP, but not unrelated compounds, to Δ*tkl1* spores, thus providing a functional connection between *TKL1*, ATP and TOR. Indeed, exogenous ATP was shown to override the specific TOR kinase inhibitor rapamycin. Although we do not know how ATP acts on TOR, these results are consistent with observations in mammalian cells, where inactivated mTOR promotes cellular quiescence while mTOR activation is dependent on the monitoring of ATP levels, either directly [42] or via the AMP-sensing TOR regulator AMPK [43]. Therefore, one explanation for the results presented here could be that, following the transition of the fungus into the host cell, *TKL1* is required to metabolize glucose and provide an ATP signal that mediates a metabolic checkpoint - via TOR - in order to control the cell cycle and promote hyphal growth *in planta*. Metabolic checkpoints sense metabolites to regulate cellular functions and have recently been implicated in cell cycle quiescence in hematopoietic stem cells [44]. However, although TOR can control quiescence in yeast [33], [34], Δ*tkl1* strains are likely in a slow growing rather than quiescent state (Figure 3F and 3G). Moreover, in addition to extending G_1_, inactivating TOR can affect the G_2_/M transition, resulting in G_2_-delay in yeast [33], [45]. Therefore, we cannot state at this time where in the cell cycle of *M. oryzae* the TOR pathway regulates mitosis in response to ATP, except that its abrogation results in mitotic delay.

Additional evidence that *TKL1* mediates an *in planta*-specific metabolic checkpoint in *M. oryzae* comes from the observations that outside the host plant, *TKL1* was not required for cell cycle regulation or autophagic cell death of the spore during appressorium formation and rice leaf penetration. Instead, *TKL1* (or exogenous ATP treatment of Δ*tkl1* strains) was required to maintain a punctate appressorial nucleus and ensure the correct timing of nuclear migration from the appressorium into the primary hyphae in the plant. H1:RFP was not localized to a punctate nuclear structure in Δ*tkl1* H1:RFP strains following host penetration at 21 hpi and was instead diffused throughout the appressorium. In fibroblasts, different histone H1 subtypes have different cellular localizations during cell division. For example, Histone H1.2 is associated with chromatin during prophase but cytoplasmically localized during metaphase and early anaphase [46]. Histone H1.5 is partitioned between chromatin and cytoplasm during metaphase and early anaphase [46]. In addition, histone H1 diffusion dynamics in HeLa cells are affected by ATP depletion [47]. Thus, H1:RFP might be localized throughout the appressorium in Δ*tkl1* H1:RFP strains at 21 hpi due to altered cell cycle progression and/or altered histone dynamics in response to ATP (compared to Guy11 H1:RFP at 21 hpi). This hypothesis remains to be tested.

In Δ*tkl1* strains in the absence exogenous ATP, at least one nucleus has migrated into the IH of Δ*tkl1* mutant strains by at least 32 hpi (Figure 5), suggesting the *TKL1*-dependent metabolic checkpoint is reversible and perhaps responds to other metabolites. Reversibility is a hallmark of slow growth and quiescence and distinguishes it from other non-growing cell states such as senescence, apoptosis or terminal differentiation [31].

Taken together, our data suggests the following testable model of infection. Appressorial formation occurs under the nutrient-starvation conditions found on the host surface and requires autophagic cell death of the spore and recycling of the spore contents into the incipient appressorium [8]. β-oxidation and the glyoxylate cycle are the dominant metabolic pathways during this stage of development [25]. A single mitotic cell cycle event occurs in the germ tube and one daughter nucleus migrates into the incipient appressorium [9]. The remaining conidial nuclei are degraded during autophagy [8], resulting in a sole appressorial nucleus. Following host penetration, and perhaps in response to G6P sensing by Tps1 [25], *M. oryzae* metabolism switches to glucose metabolism through the PPP and transketolase, resulting in NADPH production for redox and ATP production for satisfying the energetic demands of the growing fungus. ATP is also a signal, likely acting via TOR pathway activation, to control the migration of the appressorial nucleus into primary hyphae in the host cell. Once IH have developed in the host cell, transketolase is further required to propagate the ATP signal and, via TOR pathway activation, prevent delayed mitotic progression in order to permit vigorous cell-to-cell growth *in planta*. Deleting *TKL1* or treating spores with the non-hydrolysable ATP analogue AMP-PNP does not impact appressorium development but subsequent infection steps are delayed or abolished.

The work presented here has highlighted mechanisms controlling the transition from appressorial development to biotrophic growth in rice cells, but important questions remain. We wish to understand 1) whether ATP affects TOR signaling due to a direct interaction of ATP and the Tor1 kinase, or indirectly due to an interaction between ATP and additional TOR interacting protein(s) or pathways; 2) how the germ tube mitotic cell cycle event occurs during autophagy when TOR signaling is presumably inactive, whereas nuclear division in IH requires an activated TOR pathway; 3) what is the relationship between autophagy in the spore, G6P sensing by Tps1 *in planta*, and the TOR signaling pathway; 4) how does G6P sensing by Tps1 switch carbon metabolism from β-oxidation in mitochondria and peroxisomes to glucose metabolism through the PPP in the cytoplasm. Addressing these points will require further, detailed explorations of the biology of *M. oryzae* biotrophy.

In addition to shedding new light on the metabolic strategies governing rice infection, the characterizations of *TKL1* function in *M. oryzae* might also have important clinical relevance. Metabolizing glucose through the PPP and transketolase to generate NADPH is a metabolic strategy observed in cancer cells [48], activated macrophages [49] and the growth of T cells in response to pathogen challenge [50], [51]. Similar to our observations with *M. oryzae*, the transition of naive T cells to actively growing T cells is accompanied by a metabolic shift from β-oxidation to glucose utilization via the PPP [50], [51]. Moreover, transketolase, which controls the PPP along with G6PDH [52], is upregulated in certain tumors [53] and has been implicated in metastasis [52]. Transketolase inhibitors have been shown to reduce the rate of proliferation of pancreatic adenocarcinoma cells in culture [54] while, conversely, stimulating transketolase activity in cancer cells using thiamine promoted tumor growth in mice [55]. Thus, switching to glucose utilization via transketolase might be a conserved feature of proliferating cells. Consequently, small-molecule inhibitors of transketolase, such as those developed as anti-cancer therapies [54], [56], could hold promise for combating recalcitrant intracellular fungal pathogens.

The work presented here also contributes to our understanding of metabolic checkpoints by revealing a previously unknown functional relationship between *TKL1* and TOR that provides new information on the regulation of the TOR pathway. This connection might have importance beyond the field of plant pathogenicity due to the role activated TOR plays in some cancers and other serious disorders such as cardiovascular disease [57]. Moreover, metabolic checkpoints are required for T cell differentiation and immune responses [51], and this requires signaling through a metabolic checkpoint mediated by the mTOR pathway [51].

In conclusion, this study gives fresh insights into the metabolic strategies controlling, and committing, intracellular pathogens to biotrophic growth. This knowledge is likely applicable to a wide range of important fungal pathogens. Moreover, our characterization of transketolase as a metabolic checkpoint mediator might inform other areas of biology where this enzyme and its downstream targets are important, such as during cancer cell proliferation.

## Materials and Methods

### *Ex planta* culture conditions and physiological analyses

All strains used in this study are stored in the Wilson lab. Guy11 was used as the wild-type isolate [58] and all mutant strains described in this study were generated from the Guy11 parental strain (Table S1). *M. oryzae* was cultured and stored using standard procedures [37]. Strains were grown on complete medium (CM) or Cove's minimal media (MM), as described previously [59]. Glucose was used in MM at a final concentration of 1% w/v. Nitrogen sources were used in MM at a final concentration of 10 mM. Physiological analyses were performed on media as described previously [24]. Plates were imaged with a Sony Cyber-shot digital camera, 14.1 mega pixels, after 10 days of growth. For sporulation rates, strains were grown on at least three independent CM plates for 12 days before the spores were harvested and spore concentrations were measured using a hemocytometer (Corning). Appressorium developmental assays were performed on hydrophobic microscope coverslips (Fisherbrand). Spores were harvested and diluted into 1×10^5^ spores ml^−1^ in sterile distilled water. 200 µl of spore suspension was placed on three plastic coverslips per strain and placed in a plastic box with a wet paper towel on the bottom simulating a humid chamber. After 24 hr of incubation, the number of appressoria formed from 50 spores was determined for each replicate, and an average percent value generated. The non-hydrolysable ATP analogue adenosine 5′-adenylyl imidodiphosphate (AMP-PNP) was purchased from Sigma and added to Guy11 spores to a final concentration of 5 mM. The NADPH oxidase inhibitor diphehyleneiodonium chloride was purchased from Sigma and added to Guy11 spores to a final concentration of 0.4 mM, following [60].

### Homologous gene replacement strategies

Transformation-competent protoplasts were produced as previously described [22]. Guy11 was grown in liquid CM with agitation at 150 rpm for 48 hr. Mycelia was harvested and treated with lytic enzymes (Glucanex, Sigma) for 3 hr at 29°C in order to produce protoplasts. All targeted gene deletions mentioned in this study were generated using the split marker approach in which a selectable marker replaces the native gene of interest in the Guy11 genome (following [22]). *PGI1*, and *FBP1* genes were replaced in Guy11 with the *ILV1* gene conferring resistance to sulphonyl urea [22]. *TKL1* was replaced in Guy11 with the *Bar* gene confirming resistance to bialaphos, and replaced in Guy11 H1:RFP with the *ILV1* gene conferring resistance to sulphonyl urea [22]. In all cases, primers were designed to amplify a 1 kb sequence upstream and a 1 kb sequence downstream of the gene of interest (Table S2). These primers were used in a first round PCR reaction, which amplified both the right and left flanks of the gene of interest. The thermocycler conditions for the first round were 1 min at 95°C initial denaturation, followed by 34 cycles of 95°C for 30 sec denaturation, 63°C for 30 sec annealing and 68°C for 1 min extension. A second round of PCR was conducted, in which each flanking region of the gene of interest was fused to one of two overlapping pieces of the *ILV1* or *Bar* gene. For the second round PCR, similar thermocycler conditions as first round were used except for a 3 min extension time. The resulting PCR products were transformed directly into protoplasts of *M. oryzae*. Homologous gene replacements by the *ILV1* or *Bar* resistance markers were initially selected on the basis of sulphonyl urea or bialaphhos resistance, respectively. Strains carrying homologous gene replacement of the gene of interest were identified by PCR as described by Wilson et al. [22] using the oligonucleotide primers shown in Table S2. The conditions were 2 min at 95°C initial denaturation, followed by 35 cycles of 95°C for 30 sec denaturation, 63°C for 1 min annealing and 68°C for 5 min extension. A minimum of two transformants was analyzed per gene of interest. To ensure that the observed phenotype for Δ*tkl1* mutant strains were solely the result of *TKL1* deletion, a *TKL1* complementation vector was constructed using the primers in Table S2, following the protocol outlined in [24], and introduced into Δ*tkl1* mutant strains to restore virulence.

### Rice blast pathogenicity assays

Rice blast pathogenicity assays were performed as described previously [37]. Briefly, three to four week old rice seedlings from a susceptible cultivar, CO-39, were spray inoculated with spore suspensions at a rate of 1×10^5^ spores ml^−1^ in a 0.2% gelatin (Difco) solution. Plants were placed in a growth chamber with 12 hr light/dark periods. After five days, the infected leaves were collected and scored for disease symptoms. Images of the infected leaves were taken by using an Epson Workforce scanner at a resolution of 600 dpi.

### *In planta* physiological analyses

Live-cell imaging of fungal colonization of rice epidermal cells was achieved using detached rice leaf sheaths from the susceptible cultivar CO-39. Leaf sheaths were inoculated with fungal spores (1×10^5^ spores ml^−1^ in 0.20% gelatin) in the hollow interior of the sheaths as described previously [37]. Infected sheaths were kept horizontal in a glass container with humid conditions for up to 48 hr. Starting at 24 hpi, the rice sheaths were excised and observed under a light microscope (Zeiss AxioSkop). For each strain, appressorium development from 50 spores was measured on each of three independent leaf surfaces at 24 hpi to obtain an average rate of appressorium formation. Appressorium penetration rates at 30 hpi, and IH growth rates and movement to adjacent cells at 48 hpi, were determined from fifty appressoria per treatment, repeated in triplicate, following [59]. IH growth rates were determined using a 4-point scale where 1 = IH length shorter than 10 µm with no branching; 2 = IH length is 10–20 µm with 0–2 branches; 3 = IH length is longer than 20 µm and/or with more than 2 branches within one cell; 4 = IH has spread to adjacent cells. Images were taken using a Nikon A1 laser scanning confocal mounted on a Nikon 90i compound microscope (software version: NIS Elements 4.13 Build914) at the University of Nebraska-Lincoln Microscopy Center. A 1.5 zoom Z series step (1 µm) was used in the 60× lens. Transmitted light and fluoresce for td tomato were imaged with a 561.5 nm laser. td tomato fluoresce was detected at 570–620 nm. The number of nuclei per 10 µm of IH within infected rice cells was quantified using ImageJ software (rsbweb.nih.gov/ij). For all the images, the scale was set (*Analyze-Set scale*) and a 10 µm line was drawn inside the hyphae (*Analyze-Measure*). The number of nuclei along the 10 µm line was counted for each strain at 32, 48 and 65 hpi. For each timepoint, six independent replications were analyzed.

### Gene transcript analysis

Total RNA from infected leaf tissue or fungal mycelium was extracted using the RNeasy Plant Mini Kit from Qiagen. For leaf RNA extractions, deteached rice leaf sheaths were inoculated with 1×10^5^ spores ml^−1^ of the appropriate strain, isolated at 48 hpi, frozen in liquid nitrogen and ground in a mortar with a pestle. For mycelial RNA extraction, strains were grown in CM for 48 hr before switching to Cove's minimal media containing 5 nM rapamycin, 5 mM rapamycin +5 mM ATP, or no treatment, for 16 hr following [23]. Mycelia was harvested, frozen in liquid nitrogen, and lyophilized for 24 hr. A total of 100 mg of each mycelial sample was used to perform RNA extractions. RNA from mycelia or rice leaf tissue was treated with DNase I (Invitrogen) and converted to cDNA using the qScript reagents from Quantas. The cDNA reactions were performed in 20 µl reaction volumes containing 1× of qScrip cDNA Super Mix (5×) and 1–10 µg of total treated RNA. cDNA synthesis conditions were: 5 min at 25°C, 30 min at 42°C and 5 min at 85°C. After completion of cDNA synthesis, the first reaction was diluted 5 fold for PCR amplification. The resulting cDNA was analyzed by quantitative real-time PCR (qPCR) in an Eppendorf Mastercycler ep Realplex real-time PCR system. Reactions were performed in a 25 µl reaction containing 12.5 µl 2× Quantifast SYBR Green PCR Master Mix (Qiagen), 100 nM of oligonucleotide primers and 2.5 µl (≤100 ng) of cDNA. The primer sequences are provided in Table S2. Thermocycler conditions were: 5 min at 95°C initial denaturation, followed by 40 cycles of 95°C for 30 sec denaturation, 63°C for 30 sec annealing and 72°C for 1 min extension. Gene expression of each gene was normalized against the expression levels of either the *M. oryzae* actin gene (*MoACT1*) for *in planta* analysis or the *M. oryzae* β-tubulin gene (*TUB2*) for mycelial transcript analysis, as described previously [23]. Results are the average of three technical replications and at least two biological replications.

### Nucleotide quantification

The analysis of nucleotides in mycelial samples was performed using LC-MS/MS by separation of the nucleotides using hydrophilic interaction chromatography (HILIC). Samples of ground, lyophilized mycelia were weighed at about 10 mg per sample in seal proof Eppendorf tubes and extracted at 4°C with 0.6 mL of 80% MeOH/20% H_2_O (both LC-grade, Fisher Scientific). The samples were subjected to extraction at room temperature using a Bullet Blender Homogenizer (Averill Park, NY) after the addition of ∼100 µL of zirconia beads. The samples were then centrifuged for 10 minutes, the supernatants were removed, and a second extraction with 0.4 mL of 50% MeOH/50% H_2_O was performed by the same procedure. After centrifugation of the samples, aliquots were transferred into plastic LC-vials capped with sealed septums and loaded onto the LC-MS/MS system. The latter consisted on an AbSciex 4000 Qtrap Hybrid LC-MS/MS system (Framingham, MA) operating in triple quad mode using a multiple reaction monitoring (MRM) method. The instrument was interfaced to an Agilent LC1200 which included an autosampler with the samples thermostated at 4°C. The nucleotides were separated using a Phenomenex (Torrance, CA) Luna-NH2 column with dimensions of 2×250 mm; 100 Å pore size and 5 µm particle size. The MRM transitions were monitored in positive mode following [61]. The transitions were optimized with pure nucleotide solutions by infusion of 10 µM solutions directly into the mass spectrometer, followed by final ion source optimization by loop injection of those solutions. Stock solutions of ATP were prepared and quantified by their UV/visible spectra using a Cary-100 spectrophotometer with available extinction coefficients. External calibration curves were generated for 2 transitions of the nucleotide by performing injections of serial dilutions of the stock using the HILIC column interfaced with the LC-MS/MS system. Plots of integrated areas vs. Concentration in the range from 50–1000 pmoles yielded linear fits with correlation coefficients >0.99. The amounts of nucleotide was estimated from the integration of the more predominant MRM transition by comparison with the calibration curve and normalized by the total weight of the samples. Reinjection of the samples after 24 hr yielded the same areas, indicating that no significant hydrolysis of the nucleotides was occurring during LC runs. All solvents were purchased from J.T. Baker and were of LC-MS Grade Purity.

## Supporting Information

Figure S1 **Nuclear division is not impaired in Δ** ***tkl1*** **H1:RFP vegetative hyphae following growth in CM.** (**A**) Guy11 H1:RFP and Δ*tkl1* H1:RFP strains were grown in glucose-rich, liquid CM for 48 hr, and the vegetative hyphae examined using epifluorescent microscopy. Scale bar is 10 µm. (**B**) There were no statistical differences (*Student's t-test* p≤0.05) in the number of nuclei carried by Guy11 H1:RFP and Δ*tkl1* H1:RFP vegetative hyphae following growth in CM. Values are the mean of at least three independent replicates. Error bars are SD. Bars with the same letters are not significantly different.
(TIF)

Figure S2 **Nuclear division is not impaired in Δ** ***tkl1*** **H1:RFP vegetative hyphae following growth in defined minimal media with decreasing concentrations of glucose.** Guy11 H1:RFP and Δ*tkl1* H1:RFP strains were grown for 16 h in GMM with different concentration of glucose, as indicated, and the vegetative hyphae examined using epifluorescent microscopy (*left*). Scale bar is 10 µm. No statistical differences (*Student's t-test* p>0.05) in the number of nuclei carried by Guy11 H1:RFP and Δ*tkl1* H1:RFP vegetative hyphae following growth in GMM were observed (graphs, *right*). Values are the mean of at least three independent replicates. Error bars are SD. Bars with the same letters are not significantly different.
(TIF)

Figure S3 **Measuring the affect of ATP treatment on mitosis.** The mean number of nuclei in 10 µm lengths of IH was calculated, using ImageJ, for each strain at 48 hpi (**A**) and 65 hpi (**B**). Closed bars are untreated controls, open bars are strains treated with 5 mM ATP. Values are the mean of at least six independent replications. Error bars denote SD. Bars with the same letters are not significantly different (*Student's t-test* p≤0.05).
(TIF)

Figure S4 **Mitosis and autophagy are not altered in Δ** ***tkl1*** **H1:RFP strains during appressorium formation.** Spores of Guy11 H1:RFP and Δ*tkl1* H1:RFP were applied to detached rice leaf sheaths and nuclei were observed by epifluorescence during the development of appressoria at the times indicated. Note that in our hands, germinating conidia at times before 13 hpi were washed from the detached leaf sheaths during preparation of the samples for microscopy. Thus, no images were obtained of samples before 13 hpi. Scale bar is 5 µm.
(TIF)

Table S1 ***Magnaporthe oryzae*** **strains used in this study.**
(DOCX)

Table S2 **Oligonucleotide primers used in this study.**
(DOC)
