# Supplementary figures and images for: Evidence for a Transketolase-Mediated Metabolic Checkpoint Governing Biotrophic Growth in Rice Cells by the Blast Fungus Magnaporthe oryzae
Source: PLoS Pathog. 2014 Sep 4;10(9):e1004354. doi: 10.1371/journal.ppat.1004354 (PMC4154871; doi:10.1371/journal.ppat.1004354)

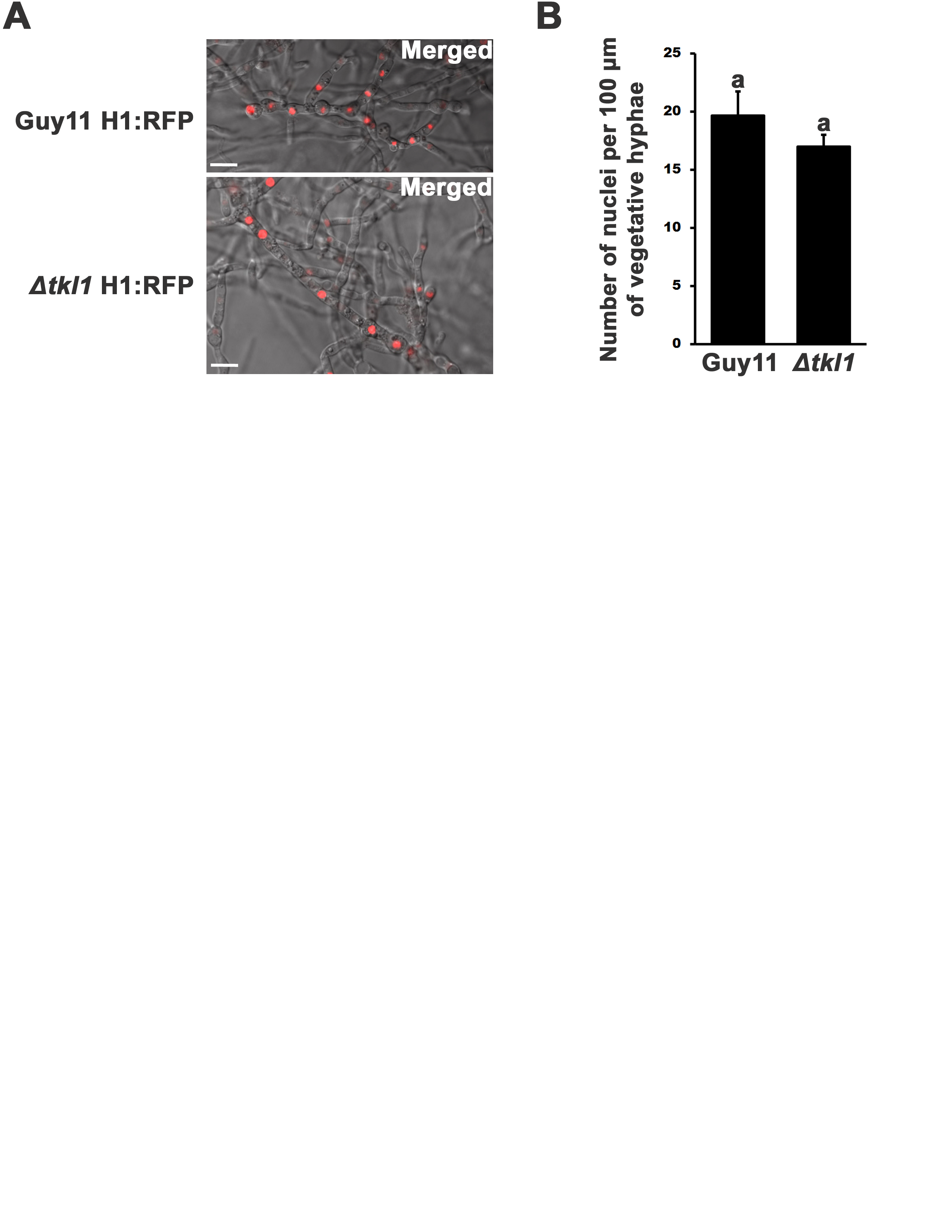

Supplement: Figure S1 — Nuclear division is not impaired in Δ tkl1 H1:RFP vegetative hyphae following growth in CM. (A) Guy11 H1:RFP and Δtkl1 H1:RFP strains were grown in glucose-rich, liquid CM for 48 hr, and the vegetative hyphae examined using epifluorescent microscopy. Scale bar is 10 µm. (B) There were no statistical differences (Student's t-test p≤0.05) in the number of nuclei carried by Guy11 H1:RFP and Δtkl1 H1:RFP vegetative hyphae following growth in CM. Values are the mean of at least three independent replicates. Error bars are SD. Bars with the same letters are not significantly different. (TIF) [file ppat.1004354.s001.tif]

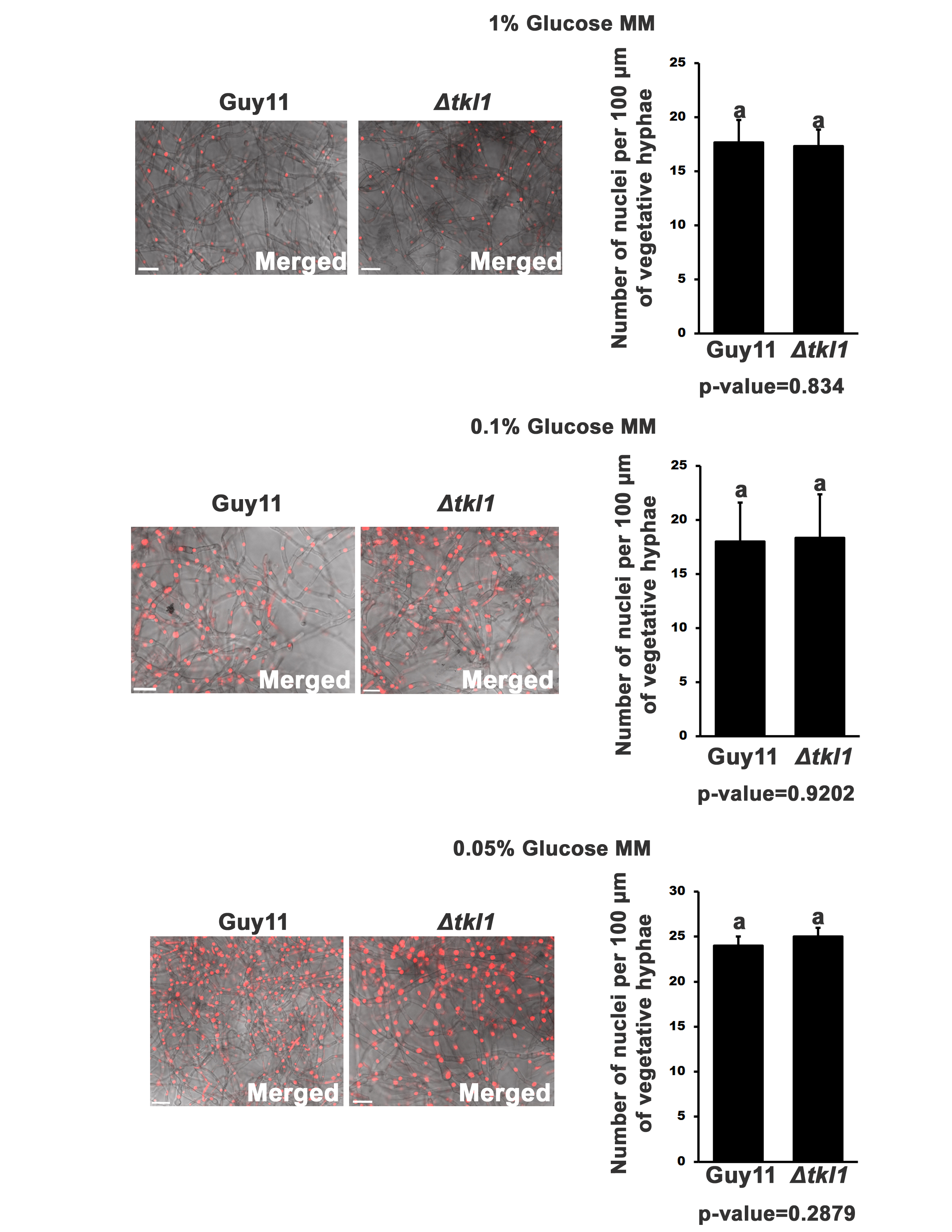

Supplement: Figure S2 — Nuclear division is not impaired in Δ tkl1 H1:RFP vegetative hyphae following growth in defined minimal media with decreasing concentrations of glucose. Guy11 H1:RFP and Δtkl1 H1:RFP strains were grown for 16 h in GMM with different concentration of glucose, as indicated, and the vegetative hyphae examined using epifluorescent microscopy (left). Scale bar is 10 µm. No statistical differences (Student's t-test p>0.05) in the number of nuclei carried by Guy11 H1:RFP and Δtkl1 H1:RFP vegetative hyphae following growth in GMM were observed (graphs, right). Values are the mean of at least three independent replicates. Error bars are SD. Bars with the same letters are not significantly different. (TIF) [file ppat.1004354.s002.tif]

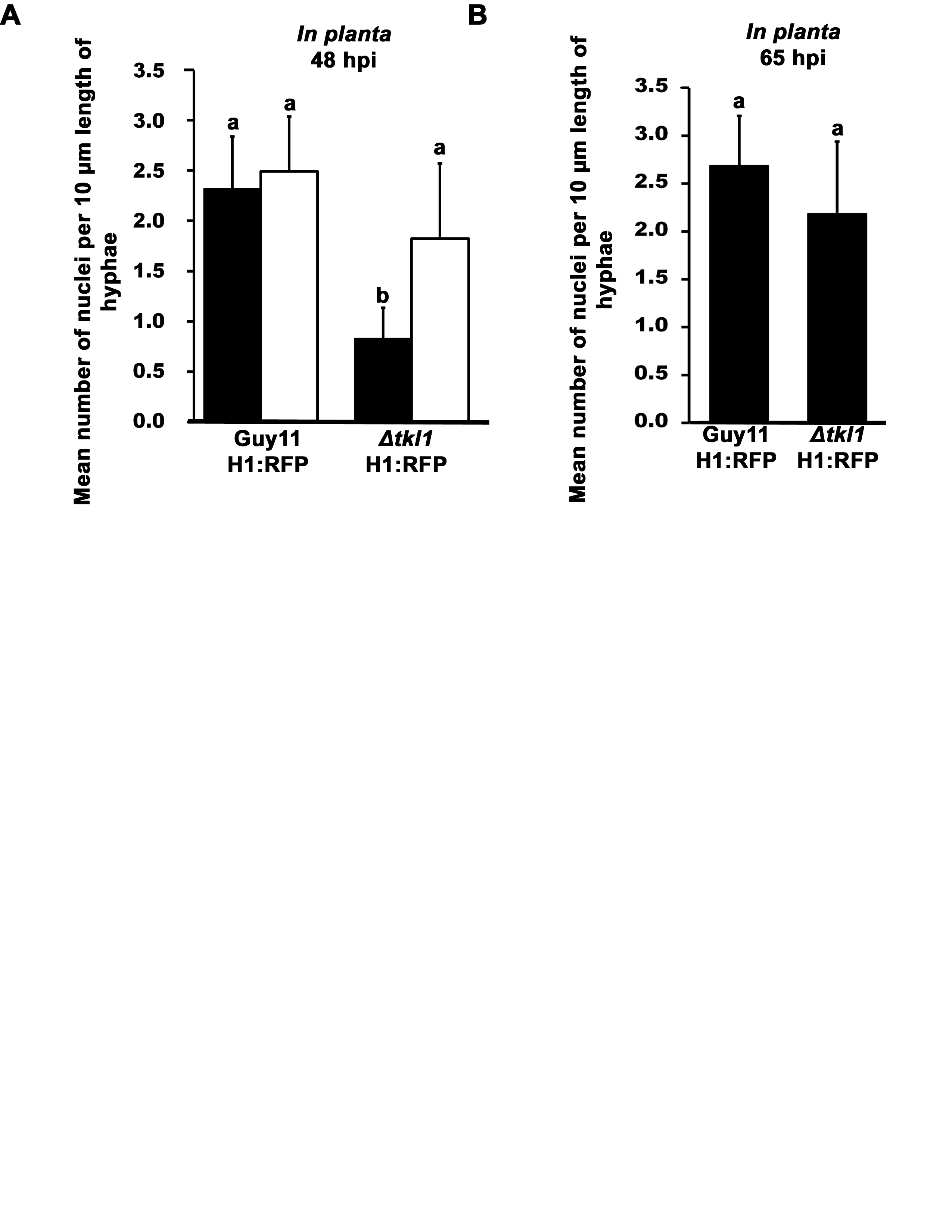

Supplement: Figure S3 — Measuring the affect of ATP treatment on mitosis. The mean number of nuclei in 10 µm lengths of IH was calculated, using ImageJ, for each strain at 48 hpi (A) and 65 hpi (B). Closed bars are untreated controls, open bars are strains treated with 5 mM ATP. Values are the mean of at least six independent replications. Error bars denote SD. Bars with the same letters are not significantly different (Student's t-test p≤0.05). (TIF) [file ppat.1004354.s003.tif]

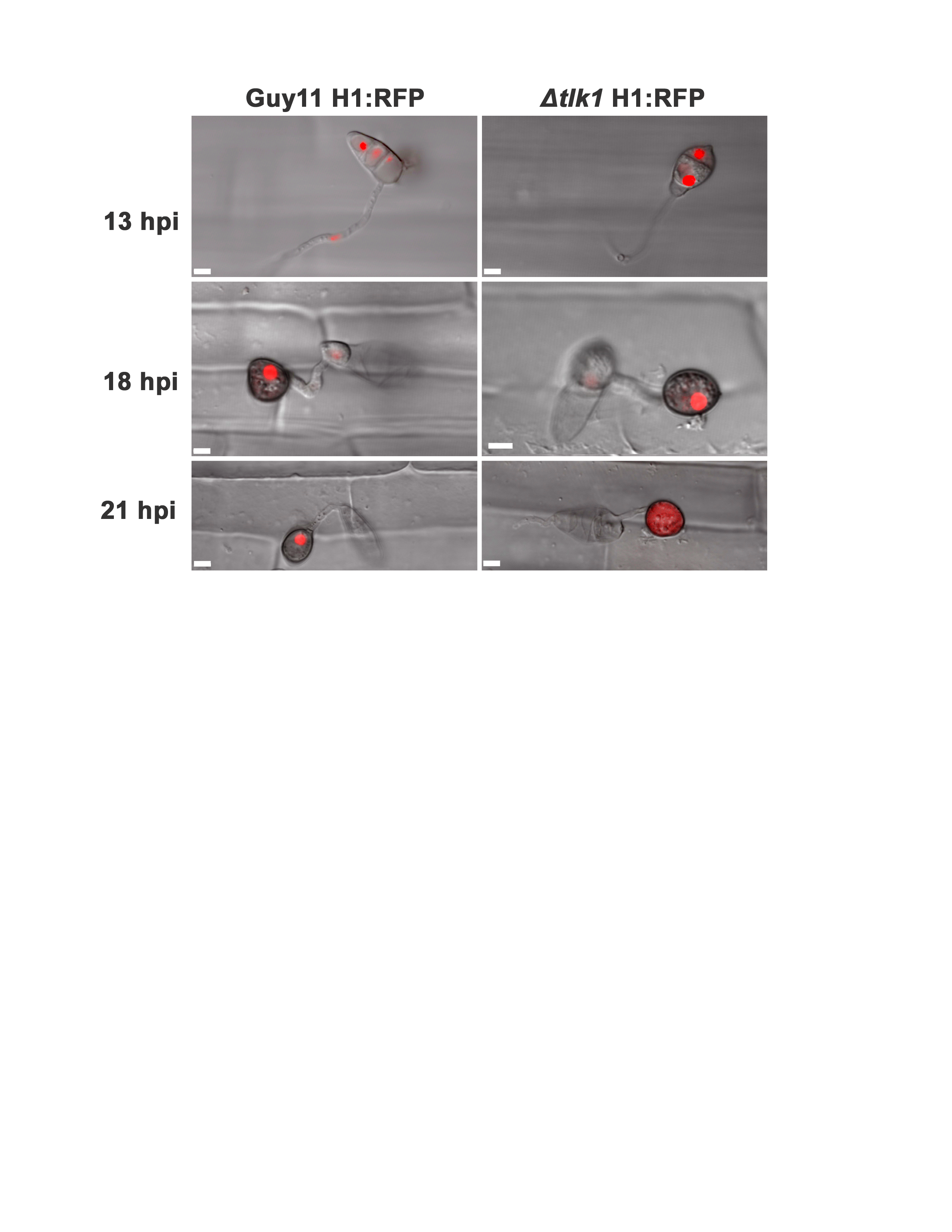

Supplement: Figure S4 — Mitosis and autophagy are not altered in Δ tkl1 H1:RFP strains during appressorium formation. Spores of Guy11 H1:RFP and Δtkl1 H1:RFP were applied to detached rice leaf sheaths and nuclei were observed by epifluorescence during the development of appressoria at the times indicated. Note that in our hands, germinating conidia at times before 13 hpi were washed from the detached leaf sheaths during preparation of the samples for microscopy. Thus, no images were obtained of samples before 13 hpi. Scale bar is 5 µm. (TIF) [file ppat.1004354.s004.tif]
