# Supplementary material for: Evidence for a Transketolase-Mediated Metabolic Checkpoint Governing Biotrophic Growth in Rice Cells by the Blast Fungus Magnaporthe oryzae
Source: PLoS Pathog. 2014 Sep 4;10(9):e1004354. doi: 10.1371/journal.ppat.1004354 (PMC4154871; doi:10.1371/journal.ppat.1004354)
Supplement: Table S1 — Magnaporthe oryzae strains used in this study. (DOCX) [file ppat.1004354.s005.docx]

**Table S1**. *Magnaporthe oryzae* strains used in this study.

| **Strains** | **Genotype** | **Reference** |
| --- | --- | --- |
| Guy11 | Wild type | [23] |
| Δ*pgi1* | Phosphoglucose isomerase (MGG_12822) deletion mutant of Guy11 | *This study* |
| Δ*fbp1* | Fructose-1,6-bisphosphate (MGG_08895) deletion mutant of Guy11 | *This study* |
| Δ*tkl1* | Transketolase (MGG_02471) deletion mutant of Guy11 | *This study* |
| Δ*tkl1 TKL1* | Complementation strain resulting from integration of the full length *TKL1* gene and native promoter into the genome of strains carrying the Δ*tkl1* gene deletion. | *This study* |
| Guy11 H1:RFP | Wild type strain expressing a histone H1 fused to a td tomato variant of red fluorescent protein | [9] |
| Δ*tkl1* H1:RFP | Transketolase (MGG_02471) deletion mutant of Guy11 H1:RFP | *This study* |
