# Supplementary material for: Evidence for a Transketolase-Mediated Metabolic Checkpoint Governing Biotrophic Growth in Rice Cells by the Blast Fungus Magnaporthe oryzae
Source: PLoS Pathog. 2014 Sep 4;10(9):e1004354. doi: 10.1371/journal.ppat.1004354 (PMC4154871; doi:10.1371/journal.ppat.1004354)
Supplement: Table S2 — Oligonucleotide primers used in this study. (DOC) [file ppat.1004354.s006.doc]

**Table S2**. Oligonucleotide primers used in this study.

| **Gene** | **Primer** | **Purpose** | **Sequence 5’ – 3’** |
| --- | --- | --- | --- |
| *ILV1* | M13F:IL | Sulphonylurea resistance gene amplification | CGCCAGGGGTTTTCCCAGTCACGACGTCGACGTGCCAACGCCACAG |
|  | ILSplit |  | AAGCATGTGCAGTGCCTTC |
|  | M13R:LV1 |  | AGCGGATAACAATTTCACACAGGAGTCGACGTGAGAGCATGCTAA |
|  | LV1Split |  | CGCCCGGCCGACATCC |
| *Bar* | M13F:BA | Bialaphos resistance gene amplification | CGCCAGGGGTTTTCCCAGTCACGACGTCGACAGAAGATGATATTGAAGGAG |
|  | BaSplit |  | GAGCCCAGTCCCGTCCG |
|  | M13R:AR |  | AGCGGATAACAATTTCACACAGGACTAAATCTCGGTGACGGGCAGG |
|  | M13F:BA |  | CGCCCGGCCGACATCC |
| *PGI1*; MGG_12822 | LF5’ | Split marker gene deletion | CCGGCGGTCAGGGAACG |
|  | LF3’ |  | GTCGTGACTGGGAAAACCCTGGCGTTTAGGCTGGTGTTGTTGTTGTGTACC |
|  | RF5’ |  | TCCTGTGTGAAATTGTTATCCGCTGAGTTGAACGTGGAGCTGCAGG |
|  | RF3’ |  | CCAGATCCGTCATTTTGCGACA |
|  | NesF |  | CTGGAAAAAGGAAGAAGACCGATT |
|  | NesR |  | GCGAGACAGATTCAACCTTCATATTTC |
| *FBP1*; MGG_08895 | LF5’ | Split marker gene deletion | GTCCTGATCAATCCGAGAGATGCTT |
|  | LF3’ |  | GTCGTGACTGGGAAAACCCTGGCGTTTTTTTTCAGAATATGACTGGCAGCTT |
|  | RF5’ |  | TCCTGTGTGAAATTGTTATCCGCTGATAGTCGCAAATCTCGCCTGAGC |
|  | RF3’ |  | GCCGACGGGACGCCTGTG |
|  | NesF |  | TGTTACACGCCGTTAATTACCATCC |
|  | NesR |  | CTCCGACGGGGCCGC |
| *TKL1*; MGG_02471 | LF5’ | Split marker gene deletion | GTTACTATGTAGGCCGACAAAAGGACG |
|  | LF3’ |  | GTCGTGACTGGGAAAACCCTGGCGGATTGCAGTTTTTTTGGGAGGGTATTA |
|  | RF5’ |  | TCCTGTGTGAAATTGTTATCCGCTTGGAGGCCTGCCGACGGTA |
|  | RF3’ |  | GCCAGCGACTCTAGGAGAGCCA |
|  | NesF |  | GGAGTAGGTAAAGCAGCGACAAAGG |
|  | NesR |  | CTCGCTTGGAGAAGATAGGTCGTG |
| *TUB2*; MGG_00604 | QRT-PCR b-tub F2 | Gene specific primers for qPCR | CGCGGCCTCAAGATGTCGT |
|  | QRT-PCR b-tub R2 |  | GCCTCCTCCTCGTACTCCTCTTCC |
| *MoACT1*; MGG_03982 | MgActin-U1 | Gene specific primers for qPCR | TCGACGTCCGAAAGGATCTGT |
|  | MgActin-L1 |  | ACTCCTGCTTCGAGATCCACATC |
| *OsACT2* | RiceACT-U1 | Gene specific primers for qPCR | CTGAAGAGCATCCTGTATTG |
|  | RiceACT-L1 |  | GAACCTTTCTGCTCCGATGG |
| *eIF4G;* MGG_06396 | RT-eIFG F1 | Gene specific primers for qPCR | GGAGTCGGGTGCGTCGTCA |
|  | RT-eIFG R1 |  | TTATTTCTTGTCTTTATCGCCAGTGTCAG |
| *RS2;* MGG_05673 | RS2F | Gene specific primers for qPCR | GGTTGCCTCGCCCGCTG |
|  | RS2R |  | CGCTTGCCGTCCCTGAGG |
| *RS3;* MGG_09222 | qRS3-F | Gene specific primers for qPCR | TCGTTCAGCCCGTCAGCCAA |
|  | qRS3-R |  | CTCCTGCTCCTCACCCTCACCC |
| *RGT2*; MGG_01446 | qRGT2-F | Gene specific primers for qPCR | GCTCGCCTTCGCCTACTTCCT |
|  | qRGT2-R |  | TCCTCCTTGGCAGACTCGGTG |
| Aspartate semi-aldehyde dehydrogenase; MGG_03051 | qASADH-F | Gene specific primers for qPCR | AATGGTGTTTGAGGAGGCGGATA |
|  | qASADH-R |  | AGAATGGATGATCCTGCTGCTCC |
| Laccase; MGG_13464 | qLacasse fw1 | Gene specific primers for qPCR | TCTTCCAGTACGAGGGTGCT |
|  | qLacasse rv1 |  | GACGGGCGAGAAGTTGATAA |
| *GAP1*; MGG_02072 | qGap1-F | Gene specific primers for qPCR | TCTGGGTCGGCGGCTGG |
|  | qGap1-R |  | TCAGCAAAAGAACCTATACAGACGCTT |
| *TOR1*; MGG_15156 | TorA-F1 | Gene specific primers for qPCR | GGGCACTGGTCGTCGGCG |
|  | TorA-R1 |  | CTACCAGAAGCTGCACCATCCAATG |
| *Nii1*; MGG_00634 | NIIF1 | Gene specific primers for qPCR | CTCTCCATCGCCACCTTTGAGG |
|  | NIIR1 |  | TCACCAATCCGGCGCG |
| *ATG8;* MGG_01062 | ATG8 R1 | Gene specific primers for qPCR | CAGGTCGCCGAAGGTGTTCTC |
|  | ATG8 F1 |  | GCCACCATCGACAAGAAGAAGTACC |
| *TKL1;* MGG_02471 | TKL1-GF | Gene-specific primers for complementation | TATAGGGCGAATTGGGTACTCAAATTGGTTGGCTGAGTCCACCGTAGATGTAAAATC |
